# A sequence variant associating with educational attainment also affects childhood cognition

**DOI:** 10.1038/srep36189

**Published:** 2016-11-04

**Authors:** Bjarni Gunnarsson, Guðrún A. Jónsdóttir, Gyða Björnsdóttir, Bettina Konte, Patrick Sulem, Snædís Kristmundsdóttir, Birte Kehr, Ómar Gústafsson, Hannes Helgason, Paul D. Iordache, Sigurgeir Ólafsson, Michael L. Frigge, Guðmar Þorleifsson, Sunna Arnarsdóttir, Berglind Stefánsdóttir, Ina Giegling, Srdjan Djurovic, Kjetil S. Sundet, Thomas Espeseth, Ingrid Melle, Annette M. Hartmann, Unnur Thorsteinsdottir, Augustine Kong, Daníel F. Guðbjartsson, Ulrich Ettinger, Ole A. Andreassen, Dan Rujescu, Jónas G. Halldórsson, Hreinn Stefánsson, Bjarni V. Halldórsson, Kári Stefánsson

**Affiliations:** 1 deCODE Genetics/Amgen, Inc., Reykjavik, Iceland; 2 Department of Psychiatry, Psychotherapy and Psychosomatics, Martin-Luther-University Halle-Wittenberg, Halle, Germany; 3 Department of Psychiatry, University of Munich (LMU), Munich, Germany; 4 School of Engineering and Natural Sciences, University of Iceland, Reykjavik, Iceland; 5 Institute of Biomedical and Neural Engineering, Reykjavík University, Reykjavík, Iceland; 6 NORMENT, KG Jebsen Centre for Psychosis Research, Department of Clinical Science, University of Bergen, Bergen, Norway; 7 Department of Medical Genetics, Oslo University Hospital, Oslo 0450, Norway; 8 Department of Psychology, University of Oslo, Oslo 0373, Norway; 9 NORMENT – KG Jebsen Centre, Institute of Clinical Medicine, University of Oslo, Oslo N-0316, Norway; 10 Faculty of Medicine, University of Iceland, Reykjavik, Iceland; 11 Department of Psychology, University of Bonn, Bonn, Germany; 12 NORMENT – KG Jebsen Centre, Division of Mental Health and Addiction, Oslo University Hospital, Oslo 0424, Norway

## Abstract

Only a few common variants in the sequence of the genome have been shown to impact cognitive traits. Here we demonstrate that polygenic scores of educational attainment predict specific aspects of childhood cognition, as measured with IQ. Recently, three sequence variants were shown to associate with educational attainment, a confluence phenotype of genetic and environmental factors contributing to academic success. We show that one of these variants associating with educational attainment, rs4851266-T, also associates with Verbal IQ in dyslexic children (***P*** = 4.3 × 10^−4^, β = 0.16 s.d.). The effect of 0.16 s.d. corresponds to 1.4 IQ points for heterozygotes and 2.8 IQ points for homozygotes. We verified this association in independent samples consisting of adults (***P*** = 8.3 × 10^−5^, β = 0.12 s.d., combined ***P*** = 2.2 x 10^−7^, β = 0.14 s.d.). Childhood cognition is unlikely to be affected by education attained later in life, and the variant explains a greater fraction of the variance in verbal IQ than in educational attainment (0.7% vs 0.12%,. ***P*** = 1.0 × 10^−5^).

Educational attainment can be conceived of as a representation of the cognitive processes necessary for progressive acquisition of knowledge and skills, collectively defined as intelligence, along with the drive of an individual in the right environment. Large genome-wide association studies have been performed (n > 400,000) on educational attainment, yielding sets of significant sequence markers. To date it has been established that some educational attainment markers are also associated with cognition^1^,^2^,^3^. However, it has not been established whether they do so through general cognitive ability (cognitive g), or through more specific forms of cognition, and the medium through which they affect educational attainment remains unexplained for the majority of them.

Intelligence quotient, IQ, is a measure of cognition known to associate with academic achievement^4^,^5^; children‘s IQ scores are predictive of their educational attainment as adults^4^,^5^,^6^. The Wechsler Intelligence Scale for Children-III (WISC-III) is a well-known measure of overall childhood intelligence, as reflected in Verbal and Performance IQ, as well as in Total IQ^7^. Performance on specific educational tasks is dependent on performance on subtests of IQ to a varying degree^8^, despite high correlation between the subtests^4^. When groups of individuals with low Performance IQ and high Verbal IQ were tested against groups of high Performance IQ and low Verbal IQ, the former had a consistently better outcome on education-related tasks^8^. The contributions of sequence markers to the subtypes of IQ may similarly vary; a recent study found a marker having a significant effect on Verbal IQ as well as reading disability and language impairment, but neither on Performance IQ nor Total IQ^9^. Hence, examining the impact of reported educational attainment markers within cohorts carefully characterised for these phenotypes, Total IQ, Verbal IQ and Performance IQ, may explain precisely how sequence markers affect educational attainment.

We studied the effect of educational attainment markers on cognition in dyslexics. Dyslexics have a learning difficulty with characteristic features of difficulties in phonological awareness, verbal memory and verbal processing speed^10^. The sequence variants that affect educational attainment in dyslexia may or may not be the ones that do so in the non-dyslexic population. One possibility is that factors related to intelligence that are somewhat important in the general population become more so in the dyslexic population^11^.

## Results

We obtained data from the Social Sciences Genetics Association Consortium (SSGAC) study on educational attainment^3^ without Icelandic participants (n = 278,948), and computed a polygenic score^12^,^13^ (PGS). We used the PGS to predict educational attainment in an independent Icelandic sample (n = 44,294). The PGS correlated with observed educational attainment scores (*P* = 4.0 × 10^−319^) and accounted for 3.2% of the variance (cf. SI Table 1).

In order to search for markers that associate with cognition we used a discovery sample consisting of 1,626 children (mean age 12.7, s.d. 3.6 years). These children had been referred to a psychologist because of learning difficulties, of whom 1,419 (87%) were diagnosed as dyslexic. In this sample, the educational attainment PGS from the SSGAC study correlated with Total IQ (2.2% of variance *P* = 1.2 × 10^−5^), Performance IQ (0.8% of variance, *P* = 7.1 × 10^−3^) and Verbal IQ (2.7% of variance, *P* = 1.9 × 10^−6^). This corroborates previous results^14^,^15^ demonstrating a substantial correlation between education and cognition phenotypes, and further substantiates that this relationship is at least in part due to genetics.

**Figure 1:**
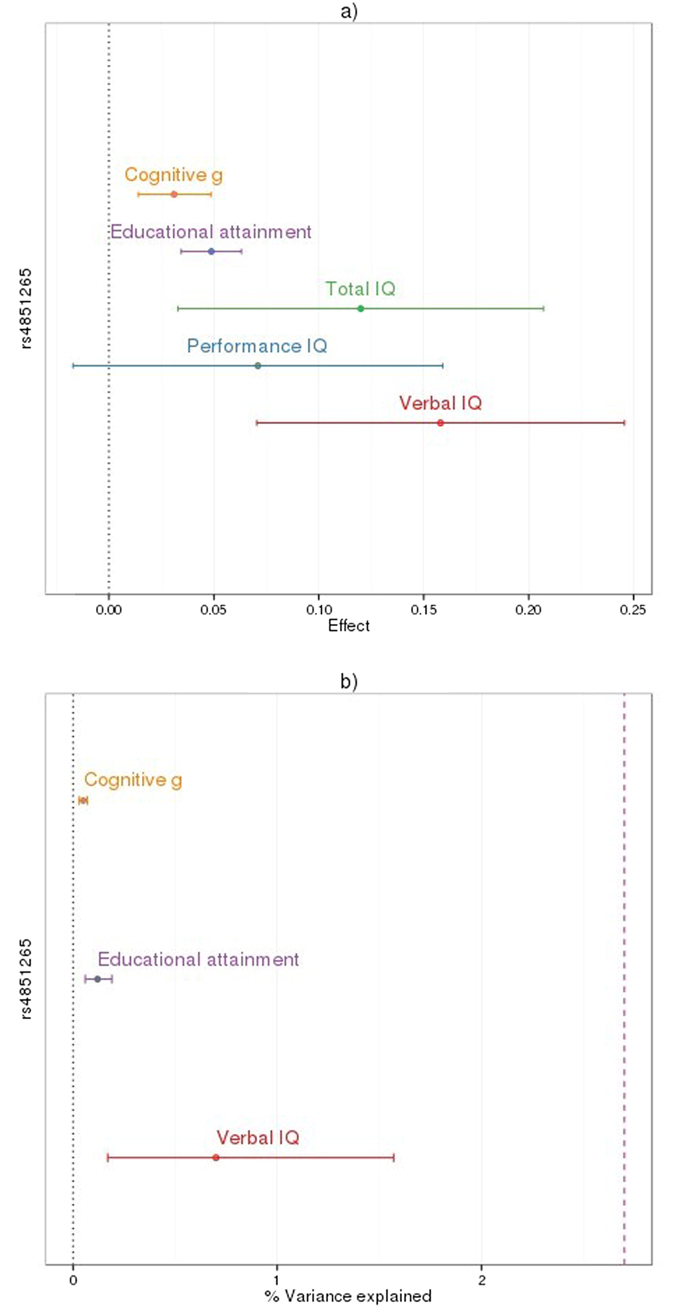
(**a**) Effects plot for rs4851266-T on WISC IQ phenotypes and educational attainment. Error bars represent 95% CI. (**b**) Variance explained plot (using replication estimates), red line shows variance explained by the polygenic score of educational attainment for Verbal IQ. Error bars represent 95% CI.

An earlier study of the SSGAC^16^ found three genome-wide significant markers for educational attainment, rs4851266, rs9320913 and rs11584700. We used these three SNPs as candidates in an association with the previously mentioned cognition phenotypes. We determined a Bonferroni-corrected threshold for significance (*P* = 0.05/3 (markers) /3 (phenotypes) = 5.6 × 10^−3^). The marker rs4851266 (2q11.2. *P* = 4.3 × 10^−4^) significantly associates with Verbal IQ (cf. Fig. 1) i.e. the allele that associates with an increase in educational attainment likewise associates with an increase in Verbal IQ in dyslexic children. Results for the three markers are listed in SI Table 2, results for 74 markers found by the SSGAC in an expanded study^3^ are further listed in SI Table 4.

**Figure 2:**
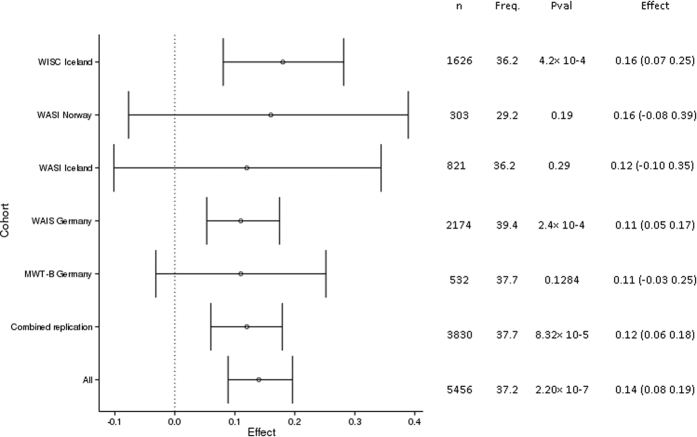
Forest plot for the GWS Verbal IQ marker (rs4851266-T) in all samples, WISC: Wechsler Intelligence Scale for Children, WAIS: Wechsler Adult Intelligence Scale, MWT-B: Mehrfachwahl-Wortschatz-Intelligenztest (Verbal IQ test),WASI: Wechsler Abbreviated Scale of Intelligence.

We followed up the association of rs4851266 in four independent adult samples (Table 1). The marker was significantly replicated with a combined *P* = 8.3 × 10^−5^ (n = 3,830) and a combined discovery and follow up *P* = 2.2 × 10^−7^ (n = 5,456), using sample-size weighting^17^,^18^ (cf. Fig. 2 Forest plot for the GWS Verbal IQ marker (rs4851266-T) in all samples, WISC: Wechsler Intelligence Scale for Children, WAIS: Wechsler Adult Intelligence Scale, MWT-B: Mehrfachwahl-Wortschatz-Intelligenztest (Verbal IQ test),WASI: Wechsler Abbreviated Scale of Intelligence.). The observed effect of the marker is consistent between samples (Cochran’s Q-test *P* = 0.56).

The Verbal IQ marker is 65 kb upstream of the *AFF3* (a.k.a. *LAF4*) gene, the only gene that is expressed in the brain that is in linkage disequilibrium with the marker^19^. The marker is known to associate with an increase in the expression of *AFF3* in the cerebellum^19^ (*P* = 3.4 × 10^−5^). *AFF3* belongs to a family of four genes that also includes *AFF1/AF4, AFF2/FMR2* and *AFF4/MCEF*. They have been shown to localize to nuclear speckles and play a role in transcription and splicing^20^,^21^. *Aff3*, the mouse homologue of *AFF3*, is expressed early in the developing mouse cortex and plays a role in cellular migration^21^.

Metsu^22^ recently mapped a folate-sensitive fragile site (FSFS) 80 kb upstream of *AFF3*, located in a brain-active alternative promoter of *AFF3*. FSFS are points on the chromosomes that tend to form gaps and break under stressful conditions in the presence of folate. Twenty-seven FSFS sites have been described cytogenetically. The best known is the FRAXA fragile site which underlies the fragile X syndrome^23^, while another is the FRAXE site that is associated with the *AFF3*-paralog *AFF2*^20^. Silencing of *AFF2* caused by this CCG trinucleotide repeat expansion results in mild intellectual disability named fragile XE or FRAXE syndrome.

Nine FSFS have been molecularly characterized to date and all are associated with a CCG/CGG trinucleotide repeat expansion. At least four of these, including *AFF3*, are associated with developmental delay^22^.

We examined the fragile site upstream of *AFF3* in our data using microsatellite genotyping^24^. We confirm that this region is highly polymorphic in the Icelandic population. Due to limitations in current sequencing technology the method used does not provide exact length of CGG repeats longer than 70 base pairs corresponding to 23.3 repeats. We find that 0.6% of the subjects possess an allele of 70 base pairs or longer, indicating a possible repeat expansion. This expansion never co-occurs with rs4851266-T in our data, and we observe an effect in Verbal IQ of −0.12 s.d. (*P* = 0.75) for the expansion.

## Discussion

These findings establish that rs4851266-T, 2q11.2, a marker previously associated with educational attainment, is associated with Verbal IQ. Consistent with these results, the *AFF3* region has previously been associated with Chinese word recognition^25^, as well as verbal-numerical reasoning^26^ and suggestively associated with cognitive g^1^,^2^,^3^. The observed effect of rs4851266-T on Verbal IQ is significantly greater than the estimated effect on educational attainment in Rietveld *et al*.^1^ (0.12 s.d. vs. 0.049 s.d., *P* = 0.022). The marker explains 0.7% (95% CI 0.17%,1.57%) of the total variance in Verbal IQ, whereas it explains only 0.12% (95% CI 0.06%, 0.19%) of the total variance in educational attainment. The large observed effect can potentially be explained by winner’s curse, however the fact that the same effect is observed consistently (Cochran’s Q-test *P* = 0.56) in our 4 replication cohorts makes this explanation unlikely.

Our results are consistent with the contribution of the marker to educational attainment being made strictly through verbal abilities; the best estimates of phenotypic correlation between IQ and educational attainment are in the range of r = 0.4–0.6^27^. We can obtain an upper bound for the effect, by assuming that the entire effect of the marker on educational attainment, 0.049 s.d., occurs through cognition. Then the estimated effect ranges between 0.057 and 0.16 for cognition, consistent with our estimate for the effect of 0.12 for Verbal IQ. Thus, the effect of the marker on Verbal IQ is sufficient to explain its effect on educational attainment. We cannot however rule out the possibility that the variant is associated with educational attainment and Verbal IQ through unmeasured traits that are correlated with both phenotypes.

This confounding effect of Verbal IQ and educational attainment is seen already in children, before the full effect of education on verbal ability has taken place. Verbal IQ is considered a measure of crystallized intelligence, a form of cognitive ability partially based on previous learning and knowledge, with verbal test performance peaking around middle age^28^. Thus, there is a reciprocal relationship between verbal ability and education. Nevertheless, an effect of the variant in such a young population (mean age of 12.7 years) suggests that it affects aspects of verbal ability that precede the effect of education.

Our results further indicate that the contribution of this locus to verbal abilities is greater than to other aspects of cognition; In Okbay *et al*.^3^, general cognitive ability (cognitive g) was used as a measure of cognition, and the variance explained by rs4851266-T for cognitive ability was significantly less than for Verbal IQ (0.7% vs 0.05%, *P* = 3.2 × 10^−3^). Cognitive g is believed to represent a general underlying cognition factor, that confounds all IQ tests and explains much of the correlation between the different subtests and test batteries. Nevertheless, despite the high correlation between cognitive processes, our results suggest that sequence variants can have differential effects on subcomponents of cognition. Future studies will hopefully highlight more markers that influence different aspects of cognition, thereby further elucidating the complex relationship between cognitive processes.

While our discovery cohort consisted mainly of dyslexic children, the results were replicated in normal adults, with a comparable effect. This suggests that the marker influences verbal abilities that are important for educational attainment, regardless of the presence of dyslexia. This may indicate that the verbal abilities affected are independent of reading per se. It confirms previous results that additive effects discovered in cohorts of dyslexia and specific language impairment can also impact the general population^29^. Thus, this demonstrates that a better understanding of the dyslexic population can provide insight into the cognitive processes of the general population. Conversely, markers important for the educational attainment of the general population also have relevance in the presence of dyslexia. In future studies it will be important to examine whether variants linked to educational attainment provide such general benefits or are specific to either the dyslexic or the general population.

Relatively few markers have been discovered that associate with cognition. This is likely due to the diversity in cognition, like in all other physiologic functions, being almost solely driven by common variants with small effects that demand larger sample sizes for discovery than has been available. Our results demonstrate the value of the large sample sizes available through composite phenotypes such as educational attainment, thereby allowing the incremental elucidation of the genetic underpinnings of complex cognitive processes.

## Methods

### Datasets

The discovery sample consisted of 1,626 Icelandic children, 1,056 males and 570 females, with a mean age at examination of 12.7 years (s.d. 3.6 years). They had been referred to a psychologist because of learning difficulties, of whom 1,419 (87%) received a diagnosis of dyslexia based on neuropsychological evaluation, 90 were diagnosed with dyscalculia (6%) and the remainder (7%) had other learning disabilities or problems of a different nature.

The follow up samples consisted of Icelandic adults (n = 821), two German samples (n = 2147, and n = 532) and a Norwegian sample (n = 303) (Table 1).

### Phenotypes

The third edition of Wechsler’s Intelligence Scale for Children^30^ (WISC-III) was administered to the Icelandic children. WISC-III contains several subtests, from which Verbal, Performance and Total IQ can be computed. In the Icelandic version^7^, Verbal IQ is comprised of the subtests Information, Similarities, Arithmetic, Vocabulary, Comprehension, whereas Performance IQ is comprised of Picture Completion, Coding, Picture Arrangement, Block Design and Object Assembly. Total IQ is calculated from all of these subtests.

In the follow up phase, an Icelandic version of the Wechsler’s Abbreviated Scale of Intelligence^31^ (WASI-II) for adults was used for the Icelanders. The WASI-II test includes four subtests: Vocabulary and Similarities, both tests of Verbal IQ, and Matrix Reasoning and Block Design, both tests of Performance IQ. A fraction of the participants were tested with an older translation of the WASI that consists of only two subtests, Vocabulary and Matrix Reasoning. WASI provides a quicker estimate of IQ using comparable subtests to the ones of WAIS, and they produce similar results^32^. For the purpose of this study, individuals with known psychiatric illnesses and copy number variations (CNVs) known to associate with psychiatric illnesses^33^ were excluded. The study was approved by the Icelandic Data Protection Authority (nr. 2001/26, with amendments) and the National Bioethics Committee, Iceland (ref. VSN_00-056, with amendments). All participating subjects signed informed consent. Personal identities of the participants and biological samples were encrypted by a third party system approved and monitored by the Icelandic Data Protection Authority.

In the first German follow up sample, the revised version of the Wechsler’s Adult Intelligence Scale^34^ (WAIS-R) was administered. This version consists of six verbal subtests, Arithmetic, Comprehension, Digit Span, Information, Similarities and Vocabulary, and five performance subtests, Block Design, Digit Symbol, Object Assembly, Picture Arrangement and Picture Completion. From these, Verbal IQ, Performance IQ and Total IQ can be calculated. Ethical approval was obtained from the University of Munich. All participants gave written informed consent.

In the second German follow up sample the MWT-B (Mehrfachwahl-Wortschatz-Intelligenztest)^35^, an established measure of Verbal IQ was used. Ethical approval was obtained from the ethics committee of the faculty of medicine of the University of Munich. All volunteers provided written informed consent and were reimbursed for their participation (25 Euros).

In the Norwegian sample the WASI scale was administered to 303 participants. Exclusion criteria was IQ below 70, hospitalized head injury, neurological disorder, unstable or uncontrolled medical condition that interferes with brain function and outside the age range of 17–65 years. All participants gave written informed consent, and the study was approved by the Regional Committee for Medical Research Ethics and the Norwegian Data Inspectorate, and the Biobank was approved by the Health Department.

All methods were carried out in accordance with relevant guidelines. The follow up results were combined using sample-size weighting (Whitlock, 2005; Zaykin, 2011).

### Genotyping

The study is based on the genotypes of 150,656 Icelanders who have been genotyped using Illumina SNP chips, along with whole-genome sequence data from 8,453 Icelanders. The whole-genome sequence data were then used for long-range phasing and imputation. For individuals that had not been genotyped with SNP chips, long range phasing and familial imputation^36^ was used to obtain their genotypes. Datasets were constructed as a part of disease association efforts at deCODE genetics. For further information regarding genotyping and imputation we refer to Gudbjartsson *et al*.^36^.

### Polygenic score

Polygenic scores (PGS) were calculated based on published data on years of education^3^ excluding Icelandic individuals and the cohort from 23andMe (n = 278,948). Only variants with MAF > 1% and info over 0.9 were used in the analysis. We used PLINK^12^,^13^ to clump variants so that variants within 500 kb window and with r2 > 0.2 with the best variant in the window were removed. PGS were calculated at 8 different P-value inclusion thresholds (P_thres∈{0.001,0.01,0.05,0.1,0.2,0.3,0.4,0.5}) and the most predictive threshold was identified at P_thres = 0.1 by comparing the significance of the association over all thresholds. At this threshold, the score was composed of 101,605 variants.

Association between PRS and quantitative traits were assessed using a linear model using the first 5 principal components of the genotypes as covariates. The predictions were controlled for age, age squared and sex. The educational attainment phenotype was corrected using the same process as in Okbay *et al*.^3^. Models were then compared using a model consisting of the covariates only (∆R^2^).

We used these markers and their score to compute a PGS score for educational attainment in our education attainment sample and the WISC-III sample. We then examined the ∆R^2^ of the computed score with observed educational attainment as well as WISC-III IQ using R^37^.

Only the individuals who were chip-typed could be used for the PGS. We therefore had 785 genotyped individuals to use for the polygenic score for WISC-IQ, and 53,995 for educational attainment.

### Proxy-phenotype method

We used a proxy-phenotype method^14^, with educational attainment as a proxy-phenotype, and the WISC IQ tests (Verbal, Performance and Total IQ) as endophenotypes. A proxy-phenotype is a phenotype sharing a genetic basis with the original for which a larger sample set exists. The method consists of identifying a set of SNPs associated with the proxy-phenotype which are then used as candidates for testing in independent samples for association with the phenotype of interest, in this case cognition.

We compared the three markers that were genome-wide significant for educational attainment^16^ with the three WISC IQ phenotypes. Markers showing a Bonferroni adjusted p-value of *P* = 0.05/9 = 5.6 × 10^−3^ or less were considered significant in the proxy-phenotype stage. All phenotypes were adjusted for sex and age, and effects are reported in units of standard deviations both in discovery and follow up samples.

Rietveld *et al*.^14^ laid down a framework for proxy-phenotype associations. In the case where the contribution towards the proxy-phenotype is strictly made through the endophenotype, the effect in the endophenotype can be estimated as


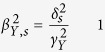


where δ_s_ is the effect of SNP s on the proxy-phenotype and γ_s_ is the phenotypic correlation between the proxy and endophenotypes.

The variance explained by a single SNP can be approximated with


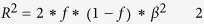


where f and β denote frequency and effect, respectively.

## Additional Information

**How to cite this article**: Gunnarsson, B. *et al*. A sequence variant associating with educational attainment also affects childhood cognition. *Sci. Rep.* **6**, 36189; doi: 10.1038/srep36189 (2016).

**Publisher’s note:** Springer Nature remains neutral with regard to jurisdictional claims in published maps and institutional affiliations.

## Supplementary Material

Supplementary Information

## Figures and Tables

**Table 1:** Nr. of individuals in samples examined; SSGAC: Social Science Genetics Association Consortium, WISC: Wechsler Intelligence Scale for Children, WAIS: Wechsler Adult Intelligence Scale, MWT-B: Mehrfachwahl-Wortschatz-Intelligenztest (Verbal IQ test), WASI: Wechsler Abbreviated Scale of Intelligence.

Discovery sample	WISC Iceland			
	N = 1,626			
Follow up samples	WAIS Germany	MWTB Germany	WASI Norway	WASI Iceland
	N = 2,174	N = 532	N = 303	N = 821
