# Supplementary material for: A sequence variant associating with educational attainment also affects childhood cognition
Source: Sci Rep. 2016 Nov 4;6:36189. doi: 10.1038/srep36189 (PMC5095652; doi:10.1038/srep36189)
Supplement: Supplementary Information [file srep36189-s1.doc]

# Supplementary Information:

# A sequence variant associating with educational attainment also affects childhood cognition

Bjarni Gunnarsson^1^*, Guðrún A. Jónsdóttir^1^, Gyða Björnsdóttir^1^, Bettina Konte^2^, Patrick Sulem^1^, Snædís Kristmundsdóttir^1^, Birte Kehr^1^, Ómar Gústafsson^1^, Hannes Helgason^1,3^, Paul D. Iordache^1,4^, Sigurgeir Ólafsson^1^, Michael L. Frigge^1^, Guðmar Þorleifsson^1^, Sunna Arnardóttir^1^, Berglind Stefánsdóttir^1^, Ina Giegling^2^, Srdjan Djurovic^5,6^, Kjetil S. Sundet^8,9^, Thomas Espeseth^8,9^, Ingrid Melle^6,8^, Annette M. Hartmann^2^, Unnur Thorsteinsdottir^1,10^, Augustine Kong^1^, Daníel F. Guðbjartsson^1,3^, Ulrich Ettinger^11^, Ole A. Andreassen^7,9^, Dan Rujescu^2^, Jónas Halldórsson^10^, Hreinn Stefánsson^1^, Bjarni V. Halldórsson^1,3^, Kári Stefánsson^1,10^ *.

Correspondence to [Bjarni.Gunnarsson@decode.is](mailto:Bjarni.Gunnarsson@decode.is), [Kari.Stefansson@decode.is](mailto:Kari.Stefansson@decode.is)

1. deCODE Genetics/Amgen, Inc., Reykjavik, Iceland.
2. Department of Psychiatry, University of Munich (LMU), Munich, Germany
3. School of Engineering and Natural Sciences, University of Iceland, Reykjavik, Iceland.
4. Institute of Biomedical and Neural Engineering, Reykjavík University, Reykjavík, Iceland.
5. NORMENT, KG Jebsen Centre for Psychosis Research, Department of Clinical Science, University of Bergen, Bergen, Norway
6. Department of Medical Genetics, Oslo University Hospital, Oslo 0450, Norway
7. NORMENT – KG Jebsen Centre, Division of Mental Health and Addiction, Oslo University Hospital, Oslo 0424, Norway.
8. Department of Psychology, University of Oslo, Oslo 0373, Norway.
9. NORMENT – KG Jebsen Centre, Institute of Clinical Medicine, University of Oslo, Oslo N-0316, Norway.
10. Faculty of Medicine, University of Iceland, Reykjavik, Iceland.
11. Department of Psychology, University of Bonn, Bonn, Germany

# Supplementary information

**Supplementary Table 1** ∆R^2^ between the polygenic score for educational attainment and phenotypes for the Icelandic discovery sample. ∆R^2^ is the difference between the variance explained in a full model including the PGS and a baseline model excluding the PGS, and the P value given is the P value for the full model.

| **Phenotype** | **Delta R2 [%]** | **P value** |
| --- | --- | --- |
| **WISC Verbal IQ** | 2.7% | 1.93E-06 |
| Arithmetic | 0.6% | 1.34E-02 |
| Comprehension | 1.7% | 1.83E-04 |
| Digit span | 0.2% | 9.07E-02 |
| Information | 1.0% | 1.22E-05 |
| Similarities | 1.0% | 3.23E-03 |
| Vocabulary | 0.8% | 7.93E-03 |
| **WISC Performance IQ** | 0.8% | 7.18E-03 |
| Block design | 1.1% | 1.91E-03 |
| Coding | 0.0% | 6.03E-01 |
| Picture completion | 0.0% | 6.83E-01 |
| Object assembly | 0.0% | 3.85E-01 |
| Picture arrangement | 0.1% | 1.70E-01 |
| **WISC Total IQ** | 2.2% | 1.27E-05 |
| **Educational attainment** | 3.2% | 3.99E-319 |

**Supplementary table 2** Genome-wide significant education markers vs cognition phenotypes, ordered by cognition phenotype p-values, sign column shows whether direction of effect is matching, + is concordant, - is discordant.

| **IQ phenotype** | **IS P** | **IS Effect** | **RS** | **Chr** | **Pos** | **A** | **MAF** | **Close genes (100 kB)** | **SSGAC P** | **SSGAC Beta** | **Sign** |
| --- | --- | --- | --- | --- | --- | --- | --- | --- | --- | --- | --- |
| Verbal IQ | 4.3E-04 | 0.158 (0.07 0.246 ) | rs4851266 | chr2 | 100202017 | T | 36.2 | LINC01104,AFF3,LONRF2,CHST10,NMS,PDCL3 | 5.33E-011 | 0.049 | + |
| Total IQ | 7.09E-03 | 0.12 (0.033 0.207 ) | rs4851266 | chr2 | 100202017 | T | 36.2 | LINC01104,AFF3,LONRF2,CHST10,NMS,PDCL3 | 5.33E-011 | 0.049 | + |
| Performance IQ | 0.104 | -0.07 (-0.154 0.014 ) | rs9320913 | chr6 | 98136857 | A | 48.9 | MIR2113,LOC101927314,POU3F2,FBXL4,MMS22L,MIR548AI | 3.50E-010 | 0.101 | - |
| Performance IQ | 0.105 | 0.086 (-0.018 0.19 ) | rs11584700 | chr1 | 204607855 | G | 19.8 | LRRN2,MDM4,TRK-TTT3-2,TRK-TTT3-1,PIK3C2B,PPP1R15B | 8.24E-012 | -0.084 | - |
| Performance IQ | 0.115 | 0.071 (-0.017 0.159 ) | rs4851266 | chr2 | 100202017 | T | 36.2 | LINC01104,AFF3,LONRF2,CHST10,NMS,PDCL3 | 5.33E-011 | 0.049 | + |
| Total IQ | 0.137 | -0.064 (-0.148 0.02 ) | rs9320913 | chr6 | 98136857 | A | 48.9 | MIR2113,LOC101927314,POU3F2,FBXL4,MMS22L,MIR548AI | 3.50E-010 | 0.101 | - |
| Total IQ | 0.422 | 0.042 (-0.061 0.145 ) | rs11584700 | chr1 | 204607855 | G | 19.8 | LRRN2,MDM4,TRK-TTT3-2,TRK-TTT3-1,PIK3C2B,PPP1R15B | 8.24E-012 | -0.084 | - |
| Verbal IQ | 0.542 | -0.026 (-0.11 0.058 ) | rs9320913 | chr6 | 98136857 | A | 48.9 | MIR2113,LOC101927314,POU3F2,FBXL4,MMS22L,MIR548AI | 3.50E-010 | 0.101 | - |
| Verbal IQ | 0.896 | -0.007 (-0.112 0.098 ) | rs11584700 | chr1 | 204607855 | G | 19.8 | LRRN2,MDM4,TRK-TTT3-2,TRK-TTT3-1,PIK3C2B,PPP1R15B | 8.24E-012 | -0.084 | + |

**Supplementary table 3** Correlations between phenotypes, correlations are all highly significant (P < 3.3 * 10^-11^)

|  | **WISC Phenotype** | **1** | **2** | **3** | **4** | **5** | **6** | **7** | **8** | **9** | **10** | **11** | **12** | **13** | **14** |
| --- | --- | --- | --- | --- | --- | --- | --- | --- | --- | --- | --- | --- | --- | --- | --- |
| **1** | **Verbal IQ** | **1** |  |  |  |  |  |  |  |  |  |  |  |  |  |
| **2** | Arithmetic | 0.520 | **1** |  |  |  |  |  |  |  |  |  |  |  |  |
| **3** | Comprehension | 0.645 | 0.438 | **1** |  |  |  |  |  |  |  |  |  |  |  |
| **4** | Digit | 0.284 | 0.416 | 0.244 | **1** |  |  |  |  |  |  |  |  |  |  |
| **5** | Information | 0.623 | 0.537 | 0.583 | 0.327 | **1** |  |  |  |  |  |  |  |  |  |
| **6** | Similarities | 0.594 | 0.466 | 0.585 | 0.323 | 0.597 | **1** |  |  |  |  |  |  |  |  |
| **7** | Vocabulary | 0.652 | 0.397 | 0.611 | 0.315 | 0.649 | 0.612 | **1** |  |  |  |  |  |  |  |
| **8** | **Performance** | 0.531 | 0.344 | 0.353 | 0.230 | 0.291 | 0.342 | 0.349 | **1** |  |  |  |  |  |  |
| **9** | Block | 0.268 | 0.426 | 0.326 | 0.277 | 0.351 | 0.379 | 0.309 | 0.688 | **1** |  |  |  |  |  |
| **10** | Coding | 0.202 | 0.407 | 0.283 | 0.311 | 0.275 | 0.279 | 0.227 | 0.519 | 0.388 | **1** |  |  |  |  |
| **11** | Picture completion | 0.312 | 0.315 | 0.446 | 0.198 | 0.381 | 0.399 | 0.416 | 0.619 | 0.478 | 0.263 | **1** |  |  |  |
| **12** | Object | 0.155 | 0.295 | 0.292 | 0.150 | 0.269 | 0.322 | 0.285 | 0.620 | 0.619 | 0.337 | 0.462 | **1** |  |  |
| **13** | Picture arrangement | 0.257 | 0.304 | 0.351 | 0.197 | 0.349 | 0.366 | 0.370 | 0.613 | 0.412 | 0.304 | 0.481 | 0.456 | **1** |  |
| **14** | **Total** | 0.872 | 0.489 | 0.567 | 0.290 | 0.518 | 0.532 | 0.571 | 0.870 | 0.544 | 0.408 | 0.532 | 0.442 | 0.500 | **1** |


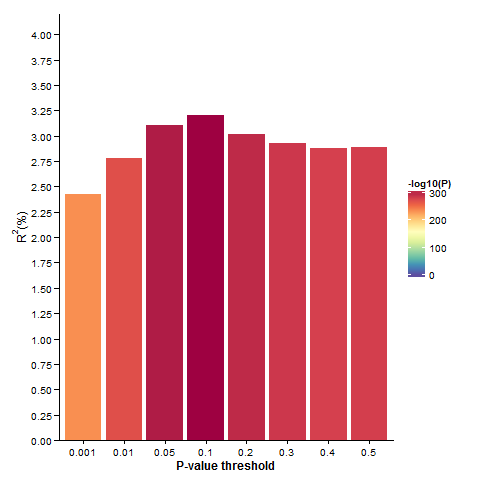


**Supplementary figure 1** R^2^ [%] for educational attainment in the Icelandic sample from the polygenic score of educational attainment for SSGAC sample.


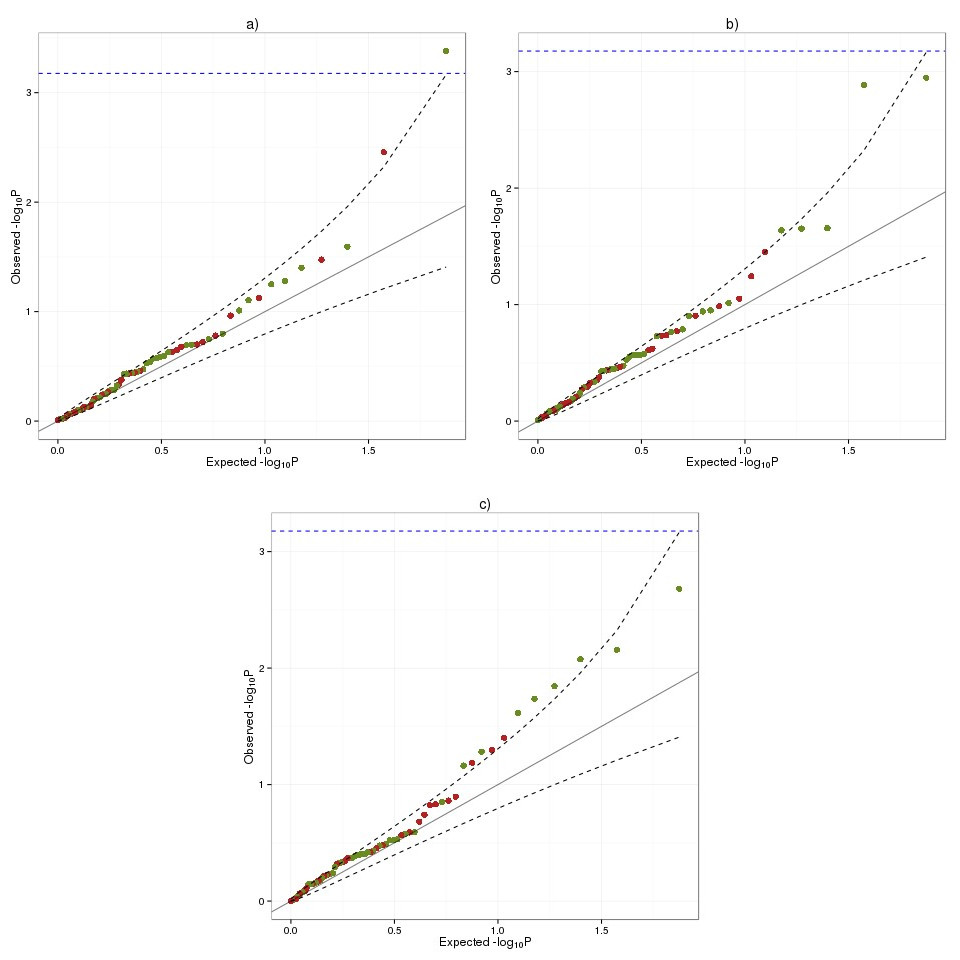


**Supplementary figure 2 a)-c)**: QQ-plots for the discovery phenotypes, a) Verbal IQ, b) Performance IQ, c) Total IQ. Colour of dots shows whether signs of effects are concordant with the original EA attainment effects, blue line represents Bonferroni threshold.

**Table 4: Proxy phenotype results for 74 markers reported by Okbay et al. 2016, sign column shows whether direction of effect is matching, + is concordant, - is discordant.**

| **IQ** | **IS P** | **IS Effect** | **RS** | **Chr** | **Pos** | **A** | **MAF** | **Close genes (100 kB)** | **SSGAC Beta** | **SSGAC P** | **Sign** |
| --- | --- | --- | --- | --- | --- | --- | --- | --- | --- | --- | --- |
| Verbal IQ | 4.2E-04 | 0.159 (0.075 0.243 ) | rs12987662 | chr2 | 100205086 | A | 36.2 | LINC01104,AFF3,LONRF2,AC092667.2 | 0.03 (0.02 0.03 ) | 2.7E-24 | + |
| Performance IQ | 1.1E-03 | 0.163 (0.065 0.261 ) | rs8005528 | chr14 | 26629405 | A | 25.2 | NOVA1-AS1,NOVA1,RP11-483C6.1 | 0.02 (0.01 0.02 ) | 7.2E-09 | + |
| Performance IQ | 1.3E-03 | -0.142 (-0.228 -0.056 ) | rs4500960 | chr2 | 161962111 | T | 47.7 | SLC4A10,DPP4,RPEP5,AC092841.1,AC092841.2 | -0.02 (-0.02 -0.01 ) | 3.8E-10 | + |
| Total IQ | 2.1E-03 | 0.154 (0.056 0.252 ) | rs8005528 | chr14 | 26629405 | A | 25.2 | NOVA1-AS1,NOVA1,RP11-483C6.1 | 0.02 (0.01 0.02 ) | 7.2E-09 | + |
| Verbal IQ | 3.5E-03 | -0.128 (-0.214 -0.042 ) | rs12531458 | chr7 | 39051098 | A | 46.6 | POU6F2,POU6F2-AS2 | 0.01 (0.01 0.02 ) | 3.1E-08 | - |
| Total IQ | 7.0E-03 | 0.12 (0.033 0.207 ) | rs12987662 | chr2 | 100205086 | A | 36.2 | LINC01104,AFF3,LONRF2,AC092667.2 | 0.03 (0.02 0.03 ) | 2.7E-24 | + |
| Total IQ | 8.4E-03 | -0.118 (-0.206 -0.03 ) | rs7955289 | chr12 | 14500733 | A | 41.4 | ATF7IP,PLBD1,RP11-502N13.2,RN7SKP134,PLBD1-AS1 | -0.02 (-0.02 -0.01 ) | 4.5E-10 | + |
| Total IQ | 1.4E-02 | -0.108 (-0.194 -0.022 ) | rs4500960 | chr2 | 161962111 | T | 47.7 | SLC4A10,DPP4,RPEP5,AC092841.1,AC092841.2 | -0.02 (-0.02 -0.01 ) | 3.8E-10 | + |
| Total IQ | 1.8E-02 | -0.11 (-0.201 -0.019 ) | rs9537821 | chr13 | 57828637 | A | 28.1 | PCDH17 | -0.02 (-0.03 -0.02 ) | 1.5E-16 | + |
| Performance IQ | 2.2E-02 | -0.107 (-0.198 -0.016 ) | rs9537821 | chr13 | 57828637 | A | 28.1 | PCDH17 | -0.02 (-0.03 -0.02 ) | 1.5E-16 | + |
| Performance IQ | 2.2E-02 | -0.103 (-0.191 -0.015 ) | rs7955289 | chr12 | 14500733 | A | 41.4 | ATF7IP,PLBD1,RP11-502N13.2,RN7SKP134,PLBD1-AS1 | -0.02 (-0.02 -0.01 ) | 4.5E-10 | + |
| Performance IQ | 2.3E-02 | 0.102 (0.014 0.19 ) | rs61160187 | chr5 | 60815752 | A | 41.2 | ELOVL7,RP11-231G3.1,ERCC8,KRT8P31,GNL3LP1 | 0.02 (0.01 0.02 ) | 3.5E-10 | + |
| Total IQ | 2.4E-02 | 0.1 (0.013 0.187 ) | rs61160187 | chr5 | 60815752 | A | 41.2 | ELOVL7,RP11-231G3.1,ERCC8,KRT8P31,GNL3LP1 | 0.02 (0.01 0.02 ) | 3.5E-10 | + |
| Verbal IQ | 2.6E-02 | 0.112 (0.014 0.21 ) | rs8005528 | chr14 | 26629405 | A | 25.2 | NOVA1-AS1,NOVA1,RP11-483C6.1 | 0.02 (0.01 0.02 ) | 7.2E-09 | + |
| Verbal IQ | 3.4E-02 | -0.094 (-0.181 -0.007 ) | rs6739979 | chr2 | 192867203 | T | 39 | PCGEM1,RPS17P8 | 0.02 (0.01 0.02 ) | 4.7E-08 | - |
| Performance IQ | 3.6E-02 | 0.097 (0.007 0.187 ) | rs12646808 | chr4 | 3248101 | T | 31.9 | MSANTD1,HTT,RGS12,RP11-357G3.2,RP11-357G3.1 | -0.02 (-0.02 -0.01 ) | 4.0E-08 | - |
| Total IQ | 4.0E-02 | 0.103 (0.005 0.201 ) | rs7767938 | chr6 | 153046478 | T | 24.9 | RGS17,AL080276.1,MTRF1L,RP1-101K10.6,FBXO5,RP1-101K10.4 | -0.02 (-0.02 -0.01 ) | 2.4E-08 | - |
| Verbal IQ | 4.0E-02 | -0.093 (-0.182 -0.004 ) | rs7955289 | chr12 | 14500733 | A | 41.4 | ATF7IP,PLBD1,RP11-502N13.2,RN7SKP134,PLBD1-AS1 | -0.02 (-0.02 -0.01 ) | 4.5E-10 | + |
| Total IQ | 5.0E-02 | -0.085 (-0.17 0 ) | rs12531458 | chr7 | 39051098 | A | 46.6 | POU6F2,POU6F2-AS2 | 0.01 (0.01 0.02 ) | 3.1E-08 | - |
| Total IQ | 5.2E-02 | 0.087 (-0.001 0.175 ) | rs2456973 | chr12 | 56023144 | A | 34.5 | IKZF4,RP11-603J24.4,RP11-603J24.21,SUOX,RPS26,RAB5B | 0.02 (0.01 0.03 ) | 1.1E-12 | + |
| Verbal IQ | 5.3E-02 | -0.101 (-0.203 0.001 ) | rs17167170 | chr7 | 133617591 | A | 22.4 | EXOC4 | -0.02 (-0.03 -0.01 ) | 1.1E-09 | + |
| Verbal IQ | 5.6E-02 | 0.086 (-0.002 0.174 ) | rs61160187 | chr5 | 60815752 | A | 41.2 | ELOVL7,RP11-231G3.1,ERCC8,KRT8P31,GNL3LP1 | 0.02 (0.01 0.02 ) | 3.5E-10 | + |
| Performance IQ | 5.7E-02 | 0.096 (-0.003 0.195 ) | rs7767938 | chr6 | 153046478 | T | 24.9 | RGS17,AL080276.1,MTRF1L,RP1-101K10.6,FBXO5,RP1-101K10.4 | -0.02 (-0.02 -0.01 ) | 2.4E-08 | - |
| Total IQ | 6.5E-02 | 0.085 (-0.005 0.175 ) | rs12646808 | chr4 | 3248101 | T | 31.9 | MSANTD1,HTT,RGS12,RP11-357G3.2,RP11-357G3.1 | -0.02 (-0.02 -0.01 ) | 4.0E-08 | - |
| Total IQ | 6.9E-02 | -0.094 (-0.195 0.007 ) | rs17167170 | chr7 | 133617591 | A | 22.4 | EXOC4 | -0.02 (-0.03 -0.01 ) | 1.1E-09 | + |
| Verbal IQ | 7.5E-02 | 0.09 (-0.009 0.189 ) | rs7767938 | chr6 | 153046478 | T | 24.9 | RGS17,AL080276.1,MTRF1L,RP1-101K10.6,FBXO5,RP1-101K10.4 | -0.02 (-0.02 -0.01 ) | 2.4E-08 | - |
| Verbal IQ | 7.9E-02 | 0.079 (-0.009 0.167 ) | rs2456973 | chr12 | 56023144 | A | 34.5 | IKZF4,RP11-603J24.4,RP11-603J24.21,SUOX,RPS26,RAB5B | 0.02 (0.01 0.03 ) | 1.1E-12 | + |
| Performance IQ | 8.9E-02 | 0.077 (-0.012 0.166 ) | rs1008078 | chr1 | 90724174 | T | 36.5 | BARHL2,RP4-665J23.1 | -0.02 (-0.02 -0.01 ) | 6.0E-10 | - |
| Performance IQ | 9.7E-02 | 0.088 (-0.016 0.192 ) | rs11588857 | chr1 | 204617919 | A | 19.6 | LRRN2,RP11-430C7.4,RP11-430C7.5,RP11-23I7.1,RNA5SP74,MDM4 | 0.02 (0.01 0.03 ) | 5.3E-10 | + |
| Verbal IQ | 9.8E-02 | -0.078 (-0.17 0.014 ) | rs9537821 | chr13 | 57828637 | A | 28.1 | PCDH17 | -0.02 (-0.03 -0.02 ) | 1.5E-16 | + |
| Performance IQ | 1.0E-01 | -0.07 (-0.154 0.014 ) | rs9320913 | chr6 | 98136857 | A | 48.9 | RP11-436D23.1,EIF4EBP2P3,RP11-111D3.2 | 0.02 (0.02 0.03 ) | 2.5E-19 | - |
| Verbal IQ | 1.1E-01 | -0.071 (-0.158 0.016 ) | rs11689269 | chr2 | 15481793 | C | 38.7 | NBAS,AC008278.2,RP11-32P22.1 | 0.02 (0.01 0.02 ) | 1.3E-08 | - |
| Performance IQ | 1.1E-01 | 0.071 (-0.017 0.159 ) | rs2456973 | chr12 | 56023144 | A | 34.5 | IKZF4,RP11-603J24.4,RP11-603J24.21,SUOX,RPS26,RAB5B | 0.02 (0.01 0.03 ) | 1.1E-12 | + |
| Performance IQ | 1.1E-01 | 0.071 (-0.017 0.159 ) | rs12987662 | chr2 | 100205086 | A | 36.2 | LINC01104,AFF3,LONRF2,AC092667.2 | 0.03 (0.02 0.03 ) | 2.7E-24 | + |
| Performance IQ | 1.2E-01 | -0.072 (-0.164 0.02 ) | rs11210860 | chr1 | 43516856 | A | 34.9 | PTPRF,HYI-AS1,HYI,SZT2,SZT2-AS1,MIR6735 | 0.02 (0.01 0.02 ) | 2.4E-10 | - |
| Performance IQ | 1.3E-01 | -0.068 (-0.155 0.019 ) | rs11712056 | chr3 | 49876964 | T | 42.1 | ACTBP13,CAMKV,MST1R,RN7SL217P,AC139451.2,TRAIP | -0.02 (-0.03 -0.02 ) | 3.3E-19 | + |
| Total IQ | 1.3E-01 | 0.068 (-0.019 0.155 ) | rs1008078 | chr1 | 90724174 | T | 36.5 | BARHL2,RP4-665J23.1 | -0.02 (-0.02 -0.01 ) | 6.0E-10 | - |
| Total IQ | 1.4E-01 | -0.064 (-0.148 0.02 ) | rs9320913 | chr6 | 98136857 | A | 48.9 | RP11-436D23.1,EIF4EBP2P3,RP11-111D3.2 | 0.02 (0.02 0.03 ) | 2.5E-19 | - |
| Total IQ | 1.4E-01 | -0.065 (-0.151 0.021 ) | rs11712056 | chr3 | 49876964 | T | 42.1 | ACTBP13,CAMKV,MST1R,RN7SL217P,AC139451.2,TRAIP | -0.02 (-0.03 -0.02 ) | 3.3E-19 | + |
| Total IQ | 1.5E-01 | -0.067 (-0.158 0.024 ) | rs4863692 | chr4 | 139842970 | T | 29.4 | MAML3,RN7SKP253 | 0.02 (0.01 0.02 ) | 1.6E-10 | - |
| Total IQ | 1.5E-01 | 0.064 (-0.023 0.151 ) | rs12682297 | chr8 | 144487477 | A | 42.2 | PPP1R16A,CTD-2517M22.14,FOXH1,KIFC2,GPT,MFSD3 | -0.02 (-0.02 -0.01 ) | 3.9E-09 | - |
| Verbal IQ | 1.6E-01 | 0.088 (-0.035 0.211 ) | rs10061788 | chr5 | 88638889 | A | 13.3 | LINC00461,MIR9-2,CTC-470C15.1,CTC-467M3.1,CTC-467M3.3,CTC-467M3.2 | 0.02 (0.01 0.03 ) | 2.5E-09 | + |
| Performance IQ | 1.6E-01 | 0.068 (-0.028 0.164 ) | rs113520408 | chr7 | 128762728 | A | 26.7 | CALU,RN7SL81P,OPN1SW,CCDC136,FAM71F1,RNA5SP243 | 0.02 (0.01 0.02 ) | 2.0E-08 | + |
| Verbal IQ | 1.7E-01 | -0.064 (-0.155 0.027 ) | rs4863692 | chr4 | 139842970 | T | 29.4 | MAML3,RN7SKP253 | 0.02 (0.01 0.02 ) | 1.6E-10 | - |
| Performance IQ | 1.7E-01 | -0.065 (-0.158 0.028 ) | rs56231335 | chr6 | 97739415 | T | 34.8 | RP1-104O17.2,RP1-104O17.3,RP1-104O17.1,RP11-436D23.1 | 0.02 (0.01 0.02 ) | 2.1E-09 | - |
| Performance IQ | 1.7E-01 | -0.058 (-0.141 0.025 ) | rs6799130 | chr3 | 161130013 | C | 48.7 | NMD3,B3GALNT1,PPM1L,RP11-479I16.2 | -0.02 (-0.02 -0.01 ) | 2.8E-08 | + |
| Verbal IQ | 1.8E-01 | -0.116 (-0.285 0.053 ) | rs112634398 | chr3 | 50038061 | A | 6.5 | RBM6,RBM5,RBM5-AS1,SEMA3F-AS1 | -0.04 (-0.05 -0.02 ) | 4.6E-08 | + |
| Total IQ | 1.8E-01 | -0.062 (-0.153 0.029 ) | rs11210860 | chr1 | 43516856 | A | 34.9 | PTPRF,HYI-AS1,HYI,SZT2,SZT2-AS1,MIR6735 | 0.02 (0.01 0.02 ) | 2.4E-10 | - |
| Performance IQ | 1.8E-01 | 0.06 (-0.028 0.148 ) | rs12682297 | chr8 | 144487477 | A | 42.2 | PPP1R16A,CTD-2517M22.14,FOXH1,KIFC2,GPT,MFSD3 | -0.02 (-0.02 -0.01 ) | 3.9E-09 | - |
| Performance IQ | 1.9E-01 | 0.07 (-0.034 0.174 ) | rs1402025 | chr5 | 114652201 | T | 20.1 | RP11-492A10.1,CTC-313D10.1 | -0.02 (-0.02 -0.01 ) | 3.4E-08 | - |
| Performance IQ | 1.9E-01 | -0.069 (-0.171 0.033 ) | rs17167170 | chr7 | 133617591 | A | 22.4 | EXOC4 | -0.02 (-0.03 -0.01 ) | 1.1E-09 | + |
| Verbal IQ | 1.9E-01 | 0.068 (-0.034 0.17 ) | rs2568955 | chr1 | 72296486 | T | 24.1 | RPL31P12,NEGR1,GDI2P2 | -0.02 (-0.02 -0.01 ) | 1.8E-08 | - |
| Verbal IQ | 2.0E-01 | 0.057 (-0.03 0.144 ) | rs12682297 | chr8 | 144487477 | A | 42.2 | PPP1R16A,CTD-2517M22.14,FOXH1,KIFC2,GPT,MFSD3 | -0.02 (-0.02 -0.01 ) | 3.9E-09 | - |
| Verbal IQ | 2.0E-01 | -0.057 (-0.144 0.03 ) | rs12772375 | chr10 | 102322931 | T | 37.8 | GBF1,AL160011.1,NFKB2,PSD,PITX3,AL121928.1 | -0.02 (-0.02 -0.01 ) | 1.6E-08 | + |
| Verbal IQ | 2.0E-01 | 0.058 (-0.031 0.147 ) | rs2837992 | chr21 | 41248593 | T | 37.2 | BACE2,FAM3B,PLAC4,BACE2-IT1,MIR3197 | 0.02 (0.01 0.02 ) | 3.8E-08 | + |
| Total IQ | 2.1E-01 | -0.055 (-0.141 0.031 ) | rs11689269 | chr2 | 15481793 | C | 38.7 | NBAS,AC008278.2,RP11-32P22.1 | 0.02 (0.01 0.02 ) | 1.3E-08 | - |
| Verbal IQ | 2.1E-01 | -0.057 (-0.146 0.032 ) | rs2610986 | chr4 | 18035608 | T | 33.8 | LCORL | 0.02 (0.01 0.02 ) | 2.0E-08 | - |
| Verbal IQ | 2.2E-01 | 0.056 (-0.034 0.146 ) | rs12646808 | chr4 | 3248101 | T | 31.9 | MSANTD1,HTT,RGS12,RP11-357G3.2,RP11-357G3.1 | -0.02 (-0.02 -0.01 ) | 4.0E-08 | - |
| Verbal IQ | 2.3E-01 | 0.053 (-0.034 0.14 ) | rs12671937 | chr7 | 93025051 | A | 49.1 | RN7SL7P,SAMD9 | -0.02 (-0.02 -0.01 ) | 9.2E-10 | - |
| Verbal IQ | 2.4E-01 | -0.055 (-0.146 0.036 ) | rs62379838 | chr5 | 120766333 | T | 32 | CTD-2334D19.1,RNU4-69P,PRR16 | -0.02 (-0.02 -0.01 ) | 3.3E-08 | + |
| Performance IQ | 2.4E-01 | 0.052 (-0.035 0.139 ) | rs7854982 | chr9 | 121882283 | T | 41.8 | TTLL11,TTLL11-IT1,RP11-244O19.1,AL365274.1,DAB2IP | -0.02 (-0.02 -0.01 ) | 1.3E-08 | - |
| Performance IQ | 2.5E-01 | -0.05 (-0.135 0.035 ) | rs3101246 | chr4 | 42647918 | T | 38.1 | ATP8A1,RP11-109E24.2,RP11-109E24.1,CCNL2P1 | 0.02 (0.01 0.02 ) | 1.4E-08 | - |
| Verbal IQ | 2.6E-01 | 0.067 (-0.048 0.182 ) | rs114598875 | chr2 | 60749249 | A | 17.6 | PAPOLG,ATP1B3P1,RNU6-612P,RP11-416L21.1,RP11-416L21.2,LINC01185 | 0.02 (0.01 0.03 ) | 2.4E-08 | + |
| Total IQ | 2.6E-01 | 0.052 (-0.038 0.142 ) | rs148734725 | chr3 | 49369275 | A | 32.7 | RHOA,RHOA-IT1,GPX1,USP4,TCTA,AMT | 0.03 (0.02 0.03 ) | 1.4E-18 | + |
| Total IQ | 2.6E-01 | 0.05 (-0.036 0.136 ) | rs2964197 | chr5 | 58239379 | T | 47.8 | PGAM1P1 | -0.02 (-0.02 -0.01 ) | 3.0E-08 | - |
| Verbal IQ | 2.6E-01 | 0.049 (-0.036 0.134 ) | rs17824247 | chr2 | 143394970 | T | 39.3 | ARHGAP15,AC096558.1,RP11-570L15.2 | 0.02 (0.01 0.02 ) | 2.8E-09 | + |
| Performance IQ | 2.6E-01 | 0.057 (-0.043 0.157 ) | rs7306755 | chr12 | 123283382 | A | 25 | SNORA9,RNA5SP375,SBNO1,CDK2AP1,RP11-282O18.7,RP11-282O18.3 | 0.02 (0.02 0.03 ) | 1.3E-12 | + |
| Total IQ | 2.7E-01 | 0.052 (-0.04 0.144 ) | rs165633 | chr22 | 29484784 | A | 31.2 | NEFH,RFPL1S,THOC5,CTA-256D12.11,RFPL1,AC000041.10 | 0.02 (0.01 0.02 ) | 2.9E-09 | + |
| Verbal IQ | 2.7E-01 | -0.049 (-0.135 0.037 ) | rs4500960 | chr2 | 161962111 | T | 47.7 | SLC4A10,DPP4,RPEP5,AC092841.1,AC092841.2 | -0.02 (-0.02 -0.01 ) | 3.8E-10 | + |
| Verbal IQ | 2.7E-01 | -0.048 (-0.133 0.037 ) | rs11690172 | chr2 | 57159959 | A | 41.8 | AC009406.1,AC009406.2 | -0.02 (-0.02 -0.01 ) | 2.0E-08 | + |
| Performance IQ | 2.7E-01 | 0.088 (-0.069 0.245 ) | rs34305371 | chr1 | 72267927 | A | 9.23 | NEGR1,GDI2P2,RPL31P12 | 0.04 (0.03 0.04 ) | 3.8E-14 | + |
| Performance IQ | 2.7E-01 | -0.053 (-0.147 0.041 ) | rs2992632 | chr1 | 243340462 | A | 27.8 | SDCCAG8,MIR4677,FCF1P7,CEP170 | -0.02 (-0.02 -0.01 ) | 8.2E-09 | + |
| Performance IQ | 2.7E-01 | -0.11 (-0.306 0.086 ) | rs2615691 | chr7 | 23362485 | A | 4.83 | IGF2BP3,AC021876.4,SNORD65,MALSU1,AC005082.1,GPNMB | -0.04 (-0.05 -0.02 ) | 4.7E-08 | + |
| Total IQ | 2.7E-01 | 0.056 (-0.044 0.156 ) | rs2568955 | chr1 | 72296486 | T | 24.1 | RPL31P12,NEGR1,GDI2P2 | -0.02 (-0.02 -0.01 ) | 1.8E-08 | - |
| Performance IQ | 2.8E-01 | 0.05 (-0.04 0.14 ) | rs148734725 | chr3 | 49369275 | A | 32.7 | RHOA,RHOA-IT1,GPX1,USP4,TCTA,AMT | 0.03 (0.02 0.03 ) | 1.4E-18 | + |
| Verbal IQ | 2.9E-01 | 0.05 (-0.043 0.143 ) | rs165633 | chr22 | 29484784 | A | 31.2 | NEFH,RFPL1S,THOC5,CTA-256D12.11,RFPL1,AC000041.10 | 0.02 (0.01 0.02 ) | 2.9E-09 | + |
| Total IQ | 3.0E-01 | -0.047 (-0.135 0.041 ) | rs12772375 | chr10 | 102322931 | T | 37.8 | GBF1,AL160011.1,NFKB2,PSD,PITX3,AL121928.1 | -0.02 (-0.02 -0.01 ) | 1.6E-08 | + |
| Verbal IQ | 3.0E-01 | 0.045 (-0.039 0.129 ) | rs895606 | chr9 | 85388753 | A | 49.6 | NA | 0.02 (0.01 0.02 ) | 2.3E-08 | + |
| Performance IQ | 3.0E-01 | -0.047 (-0.135 0.041 ) | rs11768238 | chr7 | 135542765 | A | 32.5 | NUP205,CNOT4,SDHDP2 | -0.02 (-0.02 -0.01 ) | 9.9E-10 | + |
| Total IQ | 3.0E-01 | -0.089 (-0.257 0.079 ) | rs112634398 | chr3 | 50038061 | A | 6.5 | RBM6,RBM5,RBM5-AS1,SEMA3F-AS1 | -0.04 (-0.05 -0.02 ) | 4.6E-08 | + |
| Total IQ | 3.0E-01 | 0.059 (-0.053 0.171 ) | rs76076331 | chr2 | 10837459 | T | 18.1 | PDIA6,AC092687.4,RNU7-176P,RP11-245G13.1,RP11-245G13.2,ATP6V1C2 | 0.02 (0.01 0.03 ) | 3.6E-08 | + |
| Total IQ | 3.2E-01 | -0.048 (-0.143 0.047 ) | rs2992632 | chr1 | 243340462 | A | 27.8 | SDCCAG8,MIR4677,FCF1P7,CEP170 | -0.02 (-0.02 -0.01 ) | 8.2E-09 | + |
| Total IQ | 3.3E-01 | -0.046 (-0.139 0.047 ) | rs56231335 | chr6 | 97739415 | T | 34.8 | RP1-104O17.2,RP1-104O17.3,RP1-104O17.1,RP11-436D23.1 | 0.02 (0.01 0.02 ) | 2.1E-09 | - |
| Total IQ | 3.3E-01 | 0.047 (-0.048 0.142 ) | rs113520408 | chr7 | 128762728 | A | 26.7 | CALU,RN7SL81P,OPN1SW,CCDC136,FAM71F1,RNA5SP243 | 0.02 (0.01 0.02 ) | 2.0E-08 | + |
| Performance IQ | 3.3E-01 | 0.055 (-0.057 0.167 ) | rs76076331 | chr2 | 10837459 | T | 18.1 | PDIA6,AC092687.4,RNU7-176P,RP11-245G13.1,RP11-245G13.2,ATP6V1C2 | 0.02 (0.01 0.03 ) | 3.6E-08 | + |
| Verbal IQ | 3.4E-01 | -0.043 (-0.13 0.044 ) | rs11712056 | chr3 | 49876964 | T | 42.1 | ACTBP13,CAMKV,MST1R,RN7SL217P,AC139451.2,TRAIP | -0.02 (-0.03 -0.02 ) | 3.3E-19 | + |
| Performance IQ | 3.4E-01 | 0.042 (-0.045 0.129 ) | rs2964197 | chr5 | 58239379 | T | 47.8 | PGAM1P1 | -0.02 (-0.02 -0.01 ) | 3.0E-08 | - |
| Verbal IQ | 3.5E-01 | 0.095 (-0.103 0.293 ) | rs62259535 | chr3 | 48901619 | A | 4.06 | SLC25A20,ARIH2OS,ARIH2,PRKAR2A-AS1,PRKAR2A,RP13-131K19.1 | -0.05 (-0.06 -0.03 ) | 2.6E-09 | - |
| Performance IQ | 3.5E-01 | -0.041 (-0.127 0.045 ) | rs11689269 | chr2 | 15481793 | C | 38.7 | NBAS,AC008278.2,RP11-32P22.1 | 0.02 (0.01 0.02 ) | 1.3E-08 | - |
| Total IQ | 3.5E-01 | -0.062 (-0.193 0.069 ) | rs4493682 | chr5 | 45187922 | C | 12.6 | HCN1,RP11-357F12.1 | 0.02 (0.01 0.03 ) | 3.3E-08 | - |
| Performance IQ | 3.6E-01 | 0.041 (-0.046 0.128 ) | rs13294439 | chr9 | 23358877 | A | 41.1 | NA | 0.02 (0.02 0.03 ) | 2.2E-17 | + |
| Verbal IQ | 3.6E-01 | -0.044 (-0.137 0.049 ) | rs4851251 | chr2 | 100137028 | T | 28.6 | AFF3,AC092667.2,LINC01104 | -0.02 (-0.02 -0.01 ) | 1.9E-08 | + |
| Performance IQ | 3.6E-01 | 0.043 (-0.048 0.134 ) | rs165633 | chr22 | 29484784 | A | 31.2 | NEFH,RFPL1S,THOC5,CTA-256D12.11,RFPL1,AC000041.10 | 0.02 (0.01 0.02 ) | 2.9E-09 | + |
| Verbal IQ | 3.6E-01 | -0.061 (-0.192 0.07 ) | rs4493682 | chr5 | 45187922 | C | 12.6 | HCN1,RP11-357F12.1 | 0.02 (0.01 0.03 ) | 3.3E-08 | - |
| Verbal IQ | 3.6E-01 | 0.04 (-0.046 0.126 ) | rs13294439 | chr9 | 23358877 | A | 41.1 | NA | 0.02 (0.02 0.03 ) | 2.2E-17 | + |
| Performance IQ | 3.6E-01 | 0.04 (-0.046 0.126 ) | rs1871109 | chr9 | 1746016 | T | 44.3 | NA | 0.02 (0.01 0.02 ) | 4.4E-10 | + |
| Performance IQ | 3.7E-01 | 0.046 (-0.054 0.146 ) | rs17119973 | chr14 | 84446767 | A | 23.4 | NA | -0.02 (-0.02 -0.01 ) | 3.6E-10 | - |
| Verbal IQ | 3.7E-01 | -0.042 (-0.133 0.049 ) | rs11210860 | chr1 | 43516856 | A | 34.9 | PTPRF,HYI-AS1,HYI,SZT2,SZT2-AS1,MIR6735 | 0.02 (0.01 0.02 ) | 2.4E-10 | - |
| Verbal IQ | 3.7E-01 | 0.051 (-0.06 0.162 ) | rs76076331 | chr2 | 10837459 | T | 18.1 | PDIA6,AC092687.4,RNU7-176P,RP11-245G13.1,RP11-245G13.2,ATP6V1C2 | 0.02 (0.01 0.03 ) | 3.6E-08 | + |
| Total IQ | 3.7E-01 | 0.039 (-0.046 0.124 ) | rs1871109 | chr9 | 1746016 | T | 44.3 | NA | 0.02 (0.01 0.02 ) | 4.4E-10 | + |
| Verbal IQ | 3.7E-01 | -0.054 (-0.172 0.064 ) | rs55830725 | chr2 | 236148210 | A | 15.9 | AGAP1,GBX2,AC079135.1,ASB18,RNU1-31P,RN7SL204P | -0.02 (-0.03 -0.02 ) | 5.4E-10 | + |
| Performance IQ | 3.7E-01 | 0.04 (-0.047 0.127 ) | rs6739979 | chr2 | 192867203 | T | 39 | PCGEM1,RPS17P8 | 0.02 (0.01 0.02 ) | 4.7E-08 | + |
| Performance IQ | 3.7E-01 | -0.043 (-0.138 0.052 ) | rs4851251 | chr2 | 100137028 | T | 28.6 | AFF3,AC092667.2,LINC01104 | -0.02 (-0.02 -0.01 ) | 1.9E-08 | + |
| Total IQ | 3.8E-01 | 0.046 (-0.057 0.149 ) | rs1402025 | chr5 | 114652201 | T | 20.1 | RP11-492A10.1,CTC-313D10.1 | -0.02 (-0.02 -0.01 ) | 3.4E-08 | - |
| Total IQ | 3.8E-01 | 0.04 (-0.05 0.13 ) | rs2837992 | chr21 | 41248593 | T | 37.2 | BACE2,FAM3B,PLAC4,BACE2-IT1,MIR3197 | 0.02 (0.01 0.02 ) | 3.8E-08 | + |
| Total IQ | 3.9E-01 | 0.037 (-0.048 0.122 ) | rs13294439 | chr9 | 23358877 | A | 41.1 | NA | 0.02 (0.02 0.03 ) | 2.2E-17 | + |
| Total IQ | 4.0E-01 | 0.045 (-0.059 0.149 ) | rs11588857 | chr1 | 204617919 | A | 19.6 | LRRN2,RP11-430C7.4,RP11-430C7.5,RP11-23I7.1,RNA5SP74,MDM4 | 0.02 (0.01 0.03 ) | 5.3E-10 | + |
| Total IQ | 4.0E-01 | 0.037 (-0.049 0.123 ) | rs17824247 | chr2 | 143394970 | T | 39.3 | ARHGAP15,AC096558.1,RP11-570L15.2 | 0.02 (0.01 0.02 ) | 2.8E-09 | + |
| Total IQ | 4.0E-01 | 0.049 (-0.066 0.164 ) | rs114598875 | chr2 | 60749249 | A | 17.6 | PAPOLG,ATP1B3P1,RNU6-612P,RP11-416L21.1,RP11-416L21.2,LINC01185 | 0.02 (0.01 0.03 ) | 2.4E-08 | + |
| Total IQ | 4.1E-01 | 0.051 (-0.071 0.173 ) | rs10061788 | chr5 | 88638889 | A | 13.3 | LINC00461,MIR9-2,CTC-470C15.1,CTC-467M3.1,CTC-467M3.3,CTC-467M3.2 | 0.02 (0.01 0.03 ) | 2.5E-09 | + |
| Verbal IQ | 4.2E-01 | 0.036 (-0.051 0.123 ) | rs1008078 | chr1 | 90724174 | T | 36.5 | BARHL2,RP4-665J23.1 | -0.02 (-0.02 -0.01 ) | 6.0E-10 | - |
| Performance IQ | 4.2E-01 | -0.037 (-0.127 0.053 ) | rs4863692 | chr4 | 139842970 | T | 29.4 | MAML3,RN7SKP253 | 0.02 (0.01 0.02 ) | 1.6E-10 | - |
| Total IQ | 4.3E-01 | -0.034 (-0.118 0.05 ) | rs6799130 | chr3 | 161130013 | C | 48.7 | NMD3,B3GALNT1,PPM1L,RP11-479I16.2 | -0.02 (-0.02 -0.01 ) | 2.8E-08 | + |
| Total IQ | 4.3E-01 | -0.035 (-0.121 0.051 ) | rs11768238 | chr7 | 135542765 | A | 32.5 | NUP205,CNOT4,SDHDP2 | -0.02 (-0.02 -0.01 ) | 9.9E-10 | + |
| Total IQ | 4.3E-01 | 0.079 (-0.116 0.274 ) | rs62259535 | chr3 | 48901619 | A | 4.06 | SLC25A20,ARIH2OS,ARIH2,PRKAR2A-AS1,PRKAR2A,RP13-131K19.1 | -0.05 (-0.06 -0.03 ) | 2.6E-09 | - |
| Performance IQ | 4.5E-01 | 0.046 (-0.073 0.165 ) | rs55830725 | chr2 | 236148210 | A | 15.9 | AGAP1,GBX2,AC079135.1,ASB18,RNU1-31P,RN7SL204P | -0.02 (-0.03 -0.02 ) | 5.4E-10 | - |
| Total IQ | 4.5E-01 | -0.034 (-0.123 0.055 ) | rs2610986 | chr4 | 18035608 | T | 33.8 | LCORL | 0.02 (0.01 0.02 ) | 2.0E-08 | - |
| Performance IQ | 4.6E-01 | -0.033 (-0.121 0.055 ) | rs1043209 | chr14 | 22904777 | A | 39 | RBM23,PRMT5-AS1,PRMT5,REM2,LRP10,RP11-298I3.1 | -0.02 (-0.02 -0.01 ) | 1.8E-11 | + |
| Total IQ | 4.6E-01 | -0.032 (-0.117 0.053 ) | rs3101246 | chr4 | 42647918 | T | 38.1 | ATP8A1,RP11-109E24.2,RP11-109E24.1,CCNL2P1 | 0.02 (0.01 0.02 ) | 1.4E-08 | - |
| Verbal IQ | 4.6E-01 | 0.033 (-0.055 0.121 ) | rs2964197 | chr5 | 58239379 | T | 47.8 | PGAM1P1 | -0.02 (-0.02 -0.01 ) | 3.0E-08 | - |
| Performance IQ | 4.6E-01 | -0.032 (-0.118 0.054 ) | rs16845580 | chr2 | 161064373 | T | 39.4 | AC009313.2,AC009313.1,TANK | -0.02 (-0.02 -0.01 ) | 2.7E-09 | + |
| Total IQ | 4.7E-01 | -0.035 (-0.129 0.059 ) | rs4851251 | chr2 | 100137028 | T | 28.6 | AFF3,AC092667.2,LINC01104 | -0.02 (-0.02 -0.01 ) | 1.9E-08 | + |
| Total IQ | 4.7E-01 | -0.032 (-0.119 0.055 ) | rs7131944 | chr12 | 91765780 | A | 44.1 | RP11-1041F24.1 | -0.02 (-0.02 -0.01 ) | 9.0E-09 | + |
| Performance IQ | 4.7E-01 | 0.037 (-0.063 0.137 ) | rs2568955 | chr1 | 72296486 | T | 24.1 | RPL31P12,NEGR1,GDI2P2 | -0.02 (-0.02 -0.01 ) | 1.8E-08 | - |
| Verbal IQ | 4.7E-01 | -0.032 (-0.12 0.056 ) | rs7854982 | chr9 | 121882283 | T | 41.8 | TTLL11,TTLL11-IT1,RP11-244O19.1,AL365274.1,DAB2IP | -0.02 (-0.02 -0.01 ) | 1.3E-08 | + |
| Total IQ | 4.8E-01 | -0.031 (-0.117 0.055 ) | rs6739979 | chr2 | 192867203 | T | 39 | PCGEM1,RPS17P8 | 0.02 (0.01 0.02 ) | 4.7E-08 | - |
| Performance IQ | 5.1E-01 | 0 (0 0 ) | rs192818567 | chr17 | 43991515 | T | 23.7 | MAPT,MAPT-IT1,MAPT-AS1,SPPL2C,MGC57346-CRHR1,CRHR1 | -0.02 (-0.02 -0.01 ) | 1.5E-12 | - |
| Performance IQ | 5.1E-01 | -0.038 (-0.15 0.074 ) | rs4493682 | chr5 | 45187922 | C | 12.6 | HCN1,RP11-357F12.1 | 0.03 (0.02 0.03 ) | 3.3E-08 | - |
| Total IQ | 5.1E-01 | -0.044 (-0.174 0.086 ) | rs1043209 | chr14 | 22904777 | A | 39 | RBM23,PRMT5-AS1,PRMT5,REM2,LRP10,RP11-298I3.1 | 0.02 (0.01 0.03 ) | 1.8E-11 | + |
| Verbal IQ | 5.2E-01 | -0.03 (-0.119 0.059 ) | rs12969294 | chr18 | 37606159 | A | 36.3 | CELF4,MIR4318,RP11-19F9.1 | -0.02 (-0.02 -0.01 ) | 7.2E-09 | + |
| Performance IQ | 5.2E-01 | -0.029 (-0.116 0.058 ) | rs12772375 | chr10 | 102322931 | T | 37.8 | GBF1,AL160011.1,NFKB2,PSD,PITX3,AL121928.1 | -0.02 (-0.02 -0.01 ) | 1.6E-08 | + |
| Verbal IQ | 5.2E-01 | -0.029 (-0.116 0.058 ) | rs34072092 | chr4 | 28799599 | T | 8.07 | AC096566.1,MESTP3,RN7SL101P | -0.02 (-0.02 -0.01 ) | 3.9E-08 | + |
| Verbal IQ | 5.2E-01 | -0.052 (-0.209 0.105 ) | rs7131944 | chr12 | 91765780 | A | 44.1 | RP11-1041F24.1 | -0.02 (-0.03 -0.02 ) | 9.0E-09 | + |
| Performance IQ | 5.4E-01 | -0.028 (-0.114 0.058 ) | rs1606974 | chr2 | 51646461 | A | 13.3 | AC007682.1 | -0.02 (-0.02 -0.01 ) | 2.8E-08 | - |
| Verbal IQ | 5.4E-01 | 0.038 (-0.083 0.159 ) | rs9320913 | chr6 | 98136857 | A | 48.9 | RP11-436D23.1,EIF4EBP2P3,RP11-111D3.2 | -0.02 (-0.03 -0.01 ) | 2.5E-19 | - |
| Verbal IQ | 5.6E-01 | -0.026 (-0.11 0.058 ) | rs1871109 | chr9 | 1746016 | T | 44.3 | NA | 0.02 (0.02 0.03 ) | 4.4E-10 | + |
| Verbal IQ | 5.6E-01 | 0.025 (-0.06 0.11 ) | rs2992632 | chr1 | 243340462 | A | 27.8 | SDCCAG8,MIR4677,FCF1P7,CEP170 | 0.02 (0.01 0.02 ) | 8.2E-09 | + |
| Verbal IQ | 5.7E-01 | -0.028 (-0.123 0.067 ) | rs1402025 | chr5 | 114652201 | T | 20.1 | RP11-492A10.1,CTC-313D10.1 | -0.02 (-0.02 -0.01 ) | 3.4E-08 | - |
| Performance IQ | 5.8E-01 | 0.03 (-0.075 0.135 ) | rs7131944 | chr12 | 91765780 | A | 44.1 | RP11-1041F24.1 | -0.02 (-0.02 -0.01 ) | 9.0E-09 | + |
| Total IQ | 5.8E-01 | -0.025 (-0.113 0.063 ) | rs895606 | chr9 | 85388753 | A | 49.6 | NA | -0.02 (-0.02 -0.01 ) | 2.3E-08 | + |
| Total IQ | 5.8E-01 | 0.024 (-0.06 0.108 ) | rs34072092 | chr4 | 28799599 | T | 8.07 | AC096566.1,MESTP3,RN7SL101P | 0.02 (0.01 0.02 ) | 3.9E-08 | + |
| Total IQ | 5.9E-01 | -0.044 (-0.2 0.112 ) | rs11690172 | chr2 | 57159959 | A | 41.8 | AC009406.1,AC009406.2 | -0.02 (-0.03 -0.02 ) | 2.0E-08 | + |
| Total IQ | 6.0E-01 | -0.001 (-0.127 0.125 ) | rs192818566 | chr17 | 43991515 | T | 23.7 | MAPT,MAPT-IT1,MAPT-AS1,SPPL2C,MGC57346-CRHR1,CRHR1 | -0.02 (-0.02 -0.01 ) | 1.5E-12 | - |
| Total IQ | 6.1E-01 | -0.023 (-0.107 0.061 ) | rs34305371 | chr1 | 72267927 | A | 9.23 | NEGR1,GDI2P2,RPL31P12 | -0.02 (-0.02 -0.01 ) | 3.8E-14 | + |
| Verbal IQ | 6.1E-01 | -0.03 (-0.141 0.081 ) | rs148734725 | chr3 | 49369275 | A | 32.7 | RHOA,RHOA-IT1,GPX1,USP4,TCTA,AMT | 0.03 (0.02 0.03 ) | 1.4E-18 | + |
| Performance IQ | 6.1E-01 | 0.024 (-0.068 0.116 ) | rs13402908 | chr2 | 99716915 | T | 47.7 | AFF3 | 0.03 (0.02 0.03 ) | 1.7E-11 | - |
| Verbal IQ | 6.1E-01 | 0.182 (-0.517 0.881 ) | rs1043209 | chr14 | 22904777 | A | 39 | RBM23,PRMT5-AS1,PRMT5,REM2,LRP10,RP11-298I3.1 | -0.02 (-0.03 -0.01 ) | 1.8E-11 | + |
| Performance IQ | 6.2E-01 | -0.022 (-0.107 0.063 ) | rs324886 | chr5 | 88600784 | T | 41.8 | LINC00461,CTC-470C15.1,MIR9-2,CTC-467M3.1,CTC-467M3.3,CTC-467M3.2 | -0.02 (-0.02 -0.01 ) | 1.9E-08 | + |
| Verbal IQ | 6.2E-01 | -0.023 (-0.112 0.066 ) | rs2457660 | chr2 | 60530284 | T | 34.4 | BCL11A,AC009970.1 | -0.02 (-0.02 -0.01 ) | 7.1E-10 | - |
| Verbal IQ | 6.3E-01 | 0.022 (-0.065 0.109 ) | rs1777827 | chr1 | 211439772 | A | 40.8 | ARPC3P2,LINC00467,SNX25P1,RD3,RP11-359E8.3,TRAF5 | -0.02 (-0.02 -0.01 ) | 1.6E-08 | + |
| Total IQ | 6.3E-01 | 0.022 (-0.065 0.109 ) | rs16845580 | chr2 | 161064373 | T | 39.4 | AC009313.2,AC009313.1,TANK | 0.02 (0.01 0.02 ) | 2.7E-09 | - |
| Performance IQ | 6.4E-01 | 0.021 (-0.063 0.105 ) | rs35761247 | chr3 | 48585691 | A | 6.14 | COL7A1,MIR711,UQCRC1,UCN2,PFKFB4,TMEM89 | -0.02 (-0.02 -0.01 ) | 3.8E-08 | + |
| Performance IQ | 6.4E-01 | -0.021 (-0.107 0.065 ) | rs2245901 | chr2 | 193431569 | A | 41.9 | NA | -0.02 (-0.02 -0.01 ) | 4.5E-09 | - |
| Verbal IQ | 6.6E-01 | -0.044 (-0.228 0.14 ) | rs1606974 | chr2 | 51646461 | A | 13.3 | AC007682.1 | 0.03 (0.02 0.05 ) | 2.8E-08 | + |
| Total IQ | 6.6E-01 | -0.02 (-0.105 0.065 ) | rs62379838 | chr5 | 120766333 | T | 32 | CTD-2334D19.1,RNU4-69P,PRR16 | -0.02 (-0.02 -0.01 ) | 3.3E-08 | + |
| Total IQ | 6.7E-01 | -0.027 (-0.149 0.095 ) | rs17119973 | chr14 | 84446767 | A | 23.4 | NA | -0.02 (-0.03 -0.01 ) | 3.6E-10 | + |
| Performance IQ | 6.7E-01 | -0.02 (-0.11 0.07 ) | rs17824247 | chr2 | 143394970 | T | 39.3 | ARHGAP15,AC096558.1,RP11-570L15.2 | -0.02 (-0.02 -0.01 ) | 2.8E-09 | - |
| Total IQ | 6.9E-01 | 0.022 (-0.078 0.122 ) | rs35761247 | chr3 | 48585691 | A | 6.14 | COL7A1,MIR711,UQCRC1,UCN2,PFKFB4,TMEM89 | -0.02 (-0.02 -0.01 ) | 3.8E-08 | + |
| Performance IQ | 6.9E-01 | 0.019 (-0.069 0.107 ) | rs301800 | chr1 | 8430543 | T | 16.9 | RERE,RP5-1115A15.1,RP5-1115A15.2,AL096855.1,SLC45A1 | 0.02 (0.01 0.02 ) | 1.8E-08 | - |
| Total IQ | 7.0E-01 | -0.038 (-0.223 0.147 ) | rs2245901 | chr2 | 193431569 | A | 41.9 | NA | 0.03 (0.02 0.05 ) | 4.5E-09 | - |
| Performance IQ | 7.0E-01 | -0.023 (-0.135 0.089 ) | rs62259535 | chr3 | 48901619 | A | 4.06 | SLC25A20,ARIH2OS,ARIH2,PRKAR2A-AS1,PRKAR2A,RP13-131K19.1 | 0.02 (0.01 0.03 ) | 2.6E-09 | + |
| Performance IQ | 7.0E-01 | -0.017 (-0.103 0.069 ) | rs572016 | chr12 | 120841280 | A | 45.5 | SPPL3,ARF1P2,CLIC1P1,RPL12P33,RP11-173P15.7 | -0.02 (-0.02 -0.01 ) | 3.5E-08 | - |
| Total IQ | 7.1E-01 | 0.039 (-0.158 0.236 ) | rs2615691 | chr7 | 23362485 | A | 4.83 | IGF2BP3,AC021876.4,SNORD65,MALSU1,AC005082.1,GPNMB | -0.05 (-0.06 -0.03 ) | 4.7E-08 | - |
| Performance IQ | 7.1E-01 | 0.017 (-0.069 0.103 ) | rs12531458 | chr7 | 39051098 | A | 46.6 | POU6F2,POU6F2-AS2 | -0.01 (-0.02 -0.01 ) | 3.1E-08 | + |
| Total IQ | 7.1E-01 | -0.037 (-0.231 0.157 ) | rs12671937 | chr7 | 93025051 | A | 49.1 | RN7SL7P,SAMD9 | -0.04 (-0.05 -0.02 ) | 9.2E-10 | - |
| Total IQ | 7.1E-01 | -0.016 (-0.1 0.068 ) | rs2457660 | chr2 | 60530284 | T | 34.4 | BCL11A,AC009970.1 | 0.01 (0.01 0.02 ) | 7.1E-10 | - |
| Verbal IQ | 7.2E-01 | 0.016 (-0.069 0.101 ) | rs2615691 | chr7 | 23362485 | A | 4.83 | IGF2BP3,AC021876.4,SNORD65,MALSU1,AC005082.1,GPNMB | -0.02 (-0.02 -0.01 ) | 4.7E-08 | + |
| Total IQ | 7.2E-01 | 0.017 (-0.074 0.108 ) | rs7306755 | chr12 | 123283382 | A | 25 | SNORA9,RNA5SP375,SBNO1,CDK2AP1,RP11-282O18.7,RP11-282O18.3 | 0.02 (0.01 0.02 ) | 1.3E-12 | - |
| Total IQ | 7.2E-01 | 0.037 (-0.162 0.236 ) | rs12969294 | chr18 | 37606159 | A | 36.3 | CELF4,MIR4318,RP11-19F9.1 | -0.04 (-0.05 -0.02 ) | 7.2E-09 | + |
| Verbal IQ | 7.4E-01 | 0.121 (-0.575 0.817 ) | rs3101246 | chr4 | 42647918 | T | 38.1 | ATP8A1,RP11-109E24.2,RP11-109E24.1,CCNL2P1 | -0.02 (-0.03 -0.01 ) | 1.4E-08 | + |
| Verbal IQ | 7.4E-01 | 0.12 (-0.581 0.821 ) | rs324886 | chr5 | 88600784 | T | 41.8 | LINC00461,CTC-470C15.1,MIR9-2,CTC-467M3.1,CTC-467M3.3,CTC-467M3.2 | -0.02 (-0.03 -0.01 ) | 1.9E-08 | - |
| Verbal IQ | 7.5E-01 | -0.014 (-0.097 0.069 ) | rs7945718 | chr11 | 12727272 | A | 36.2 | TEAD1,RP11-47J17.3 | 0.02 (0.01 0.02 ) | 1.5E-08 | - |
| Performance IQ | 7.5E-01 | -0.015 (-0.105 0.075 ) | rs10496091 | chr2 | 61255126 | A | 32.8 | USP34,RP11-479F13.1,RP11-493E12.2,AHSA2,AC016747.1,C2orf74 | -0.02 (-0.02 -0.01 ) | 5.6E-10 | - |
| Performance IQ | 7.5E-01 | 0.014 (-0.071 0.099 ) | rs1777827 | chr1 | 211439772 | A | 40.8 | ARPC3P2,LINC00467,SNX25P1,RD3,RP11-359E8.3,TRAF5 | -0.02 (-0.02 -0.01 ) | 1.6E-08 | + |
| Performance IQ | 7.6E-01 | 0.014 (-0.072 0.1 ) | rs62263923 | chr3 | 85625640 | A | 38 | CADM2,AC018361.1 | -0.02 (-0.02 -0.01 ) | 7.0E-09 | - |
| Total IQ | 7.7E-01 | -0.014 (-0.1 0.072 ) | rs301800 | chr1 | 8430543 | T | 16.9 | RERE,RP5-1115A15.1,RP5-1115A15.2,AL096855.1,SLC45A1 | -0.02 (-0.02 -0.01 ) | 1.8E-08 | - |
| Verbal IQ | 7.7E-01 | 0.013 (-0.072 0.098 ) | rs13402908 | chr2 | 99716915 | T | 47.7 | AFF3 | 0.02 (0.01 0.02 ) | 1.7E-11 | + |
| Verbal IQ | 7.9E-01 | -0.017 (-0.132 0.098 ) | rs572016 | chr12 | 120841280 | A | 45.5 | SPPL3,ARF1P2,CLIC1P1,RPL12P33,RP11-173P15.7 | 0.02 (0.01 0.03 ) | 3.5E-08 | + |
| Verbal IQ | 7.9E-01 | 0.013 (-0.075 0.101 ) | rs35761247 | chr3 | 48585691 | A | 6.14 | COL7A1,MIR711,UQCRC1,UCN2,PFKFB4,TMEM89 | -0.02 (-0.02 -0.01 ) | 3.8E-08 | - |
| Verbal IQ | 8.0E-01 | -0.012 (-0.099 0.075 ) | rs11768238 | chr7 | 135542765 | A | 32.5 | NUP205,CNOT4,SDHDP2 | -0.01 (-0.02 -0.01 ) | 9.9E-10 | - |
| Performance IQ | 8.0E-01 | -0.025 (-0.209 0.159 ) | rs34072092 | chr4 | 28799599 | T | 8.07 | AC096566.1,MESTP3,RN7SL101P | 0.03 (0.02 0.05 ) | 3.9E-08 | + |
| Performance IQ | 8.0E-01 | -0.012 (-0.103 0.079 ) | rs114598875 | chr2 | 60749249 | A | 17.6 | PAPOLG,ATP1B3P1,RNU6-612P,RP11-416L21.1,RP11-416L21.2,LINC01185 | -0.02 (-0.02 -0.01 ) | 2.4E-08 | - |
| Performance IQ | 8.1E-01 | -0.02 (-0.172 0.132 ) | rs62379838 | chr5 | 120766333 | T | 32 | CTD-2334D19.1,RNU4-69P,PRR16 | -0.02 (-0.03 -0.02 ) | 3.3E-08 | + |
| Total IQ | 8.1E-01 | 0.015 (-0.1 0.13 ) | rs7945718 | chr11 | 12727272 | A | 36.2 | TEAD1,RP11-47J17.3 | 0.02 (0.01 0.03 ) | 1.5E-08 | + |
| Verbal IQ | 8.1E-01 | 0.011 (-0.079 0.101 ) | rs113520408 | chr7 | 128762728 | A | 26.7 | CALU,RN7SL81P,OPN1SW,CCDC136,FAM71F1,RNA5SP243 | -0.02 (-0.02 -0.01 ) | 2.0E-08 | + |
| Performance IQ | 8.1E-01 | 0.01 (-0.073 0.093 ) | rs2610986 | chr4 | 18035608 | T | 33.8 | LCORL | -0.02 (-0.02 -0.01 ) | 2.0E-08 | - |
| Total IQ | 8.2E-01 | 0.011 (-0.081 0.103 ) | rs7854982 | chr9 | 121882283 | T | 41.8 | TTLL11,TTLL11-IT1,RP11-244O19.1,AL365274.1,DAB2IP | 0.02 (0.01 0.02 ) | 1.3E-08 | - |
| Performance IQ | 8.3E-01 | -0.011 (-0.103 0.081 ) | rs11191193 | chr10 | 102042651 | A | 31.5 | C10orf76,HPS6,RP11-302K17.3,RP11-302K17.4,LDB1,PPRC1 | 0.02 (0.01 0.02 ) | 5.4E-11 | + |
| Performance IQ | 8.3E-01 | 0.01 (-0.075 0.095 ) | rs112634398 | chr3 | 50038061 | A | 6.5 | RBM6,RBM5,RBM5-AS1,SEMA3F-AS1 | -0.02 (-0.02 -0.01 ) | 4.6E-08 | - |
| Verbal IQ | 8.3E-01 | -0.001 (-0.111 0.109 ) | rs192818565 | chr17 | 43991515 | T | 23.7 | MAPT,MAPT-IT1,MAPT-AS1,SPPL2C,MGC57346-CRHR1,CRHR1 | -0.02 (-0.02 -0.01 ) | 1.5E-12 | - |
| Verbal IQ | 8.4E-01 | 0.01 (-0.079 0.099 ) | rs34305371 | chr1 | 72267927 | A | 9.23 | NEGR1,GDI2P2,RPL31P12 | -0.02 (-0.02 -0.01 ) | 3.8E-14 | - |
| Total IQ | 8.4E-01 | -0.019 (-0.191 0.153 ) | rs62263923 | chr3 | 85625640 | A | 38 | CADM2,AC018361.1 | -0.04 (-0.05 -0.02 ) | 7.0E-09 | + |
| Performance IQ | 8.4E-01 | -0.012 (-0.121 0.097 ) | rs10061788 | chr5 | 88638889 | A | 13.3 | LINC00461,MIR9-2,CTC-470C15.1,CTC-467M3.1,CTC-467M3.3,CTC-467M3.2 | 0.03 (0.02 0.03 ) | 2.5E-09 | - |
| Verbal IQ | 8.5E-01 | -0.017 (-0.178 0.144 ) | rs301800 | chr1 | 8430543 | T | 16.9 | RERE,RP5-1115A15.1,RP5-1115A15.2,AL096855.1,SLC45A1 | 0.04 (0.03 0.04 ) | 1.8E-08 | - |
| Total IQ | 8.5E-01 | 0.009 (-0.078 0.096 ) | rs55830725 | chr2 | 236148210 | A | 15.9 | AGAP1,GBX2,AC079135.1,ASB18,RNU1-31P,RN7SL204P | 0.02 (0.01 0.02 ) | 5.4E-10 | + |
| Verbal IQ | 8.6E-01 | 0.012 (-0.107 0.131 ) | rs7306755 | chr12 | 123283382 | A | 25 | SNORA9,RNA5SP375,SBNO1,CDK2AP1,RP11-282O18.7,RP11-282O18.3 | 0.02 (0.01 0.03 ) | 1.3E-12 | + |
| Total IQ | 8.6E-01 | -0.011 (-0.126 0.104 ) | rs11191193 | chr10 | 102042651 | A | 31.5 | C10orf76,HPS6,RP11-302K17.3,RP11-302K17.4,LDB1,PPRC1 | 0.02 (0.01 0.03 ) | 5.4E-11 | - |
| Performance IQ | 8.6E-01 | -0.011 (-0.127 0.105 ) | rs12671937 | chr7 | 93025051 | A | 49.1 | RN7SL7P,SAMD9 | -0.02 (-0.03 -0.02 ) | 9.2E-10 | + |
| Verbal IQ | 8.7E-01 | -0.009 (-0.106 0.088 ) | rs17119973 | chr14 | 84446767 | A | 23.4 | NA | 0.02 (0.02 0.03 ) | 3.6E-10 | - |
| Verbal IQ | 8.9E-01 | 0.008 (-0.082 0.098 ) | rs11191193 | chr10 | 102042651 | A | 31.5 | C10orf76,HPS6,RP11-302K17.3,RP11-302K17.4,LDB1,PPRC1 | -0.02 (-0.02 -0.01 ) | 5.4E-11 | - |
| Verbal IQ | 8.9E-01 | -0.008 (-0.1 0.084 ) | rs56231335 | chr6 | 97739415 | T | 34.8 | RP1-104O17.2,RP1-104O17.3,RP1-104O17.1,RP11-436D23.1 | -0.02 (-0.02 -0.01 ) | 2.1E-09 | + |
| Total IQ | 9.0E-01 | -0.008 (-0.103 0.087 ) | rs1606974 | chr2 | 51646461 | A | 13.3 | AC007682.1 | -0.02 (-0.02 -0.01 ) | 2.8E-08 | + |
| Performance IQ | 9.0E-01 | 0.007 (-0.091 0.105 ) | rs2837992 | chr21 | 41248593 | T | 37.2 | BACE2,FAM3B,PLAC4,BACE2-IT1,MIR3197 | -0.02 (-0.02 -0.01 ) | 3.8E-08 | - |
| Performance IQ | 9.2E-01 | -0.007 (-0.105 0.091 ) | rs895606 | chr9 | 85388753 | A | 49.6 | NA | 0.02 (0.01 0.02 ) | 2.3E-08 | - |
| Total IQ | 9.2E-01 | 0.008 (-0.122 0.138 ) | rs1777827 | chr1 | 211439772 | A | 40.8 | ARPC3P2,LINC00467,SNX25P1,RD3,RP11-359E8.3,TRAF5 | -0.02 (-0.03 -0.01 ) | 1.6E-08 | - |
| Verbal IQ | 9.3E-01 | -0.005 (-0.087 0.077 ) | rs62263923 | chr3 | 85625640 | A | 38 | CADM2,AC018361.1 | 0.02 (0.01 0.02 ) | 7.0E-09 | - |
| Performance IQ | 9.3E-01 | -0.005 (-0.098 0.088 ) | rs2457660 | chr2 | 60530284 | T | 34.4 | BCL11A,AC009970.1 | 0.02 (0.01 0.02 ) | 7.1E-10 | - |
| Performance IQ | 9.3E-01 | -0.004 (-0.084 0.076 ) | rs7945718 | chr11 | 12727272 | A | 36.2 | TEAD1,RP11-47J17.3 | -0.02 (-0.02 -0.01 ) | 1.5E-08 | + |
| Verbal IQ | 9.4E-01 | 0.004 (-0.085 0.093 ) | rs11588857 | chr1 | 204617919 | A | 19.6 | LRRN2,RP11-430C7.4,RP11-430C7.5,RP11-23I7.1,RNA5SP74,MDM4 | 0.02 (0.01 0.02 ) | 5.3E-10 | + |
| Verbal IQ | 9.5E-01 | -0.004 (-0.095 0.087 ) | rs2245901 | chr2 | 193431569 | A | 41.9 | NA | 0.02 (0.01 0.02 ) | 4.5E-09 | - |
| Verbal IQ | 9.5E-01 | 0.004 (-0.088 0.096 ) | rs2431108 | chr5 | 104612267 | T | 28.4 | RP11-6N13.1 | -0.02 (-0.02 -0.01 ) | 5.3E-09 | - |
| Verbal IQ | 9.6E-01 | -0.004 (-0.103 0.095 ) | rs16845580 | chr2 | 161064373 | T | 39.4 | AC009313.2,AC009313.1,TANK | 0.02 (0.01 0.03 ) | 2.7E-09 | - |
| Total IQ | 9.6E-01 | 0.003 (-0.097 0.103 ) | rs13402908 | chr2 | 99716915 | T | 47.7 | AFF3 | -0.02 (-0.02 -0.01 ) | 1.7E-11 | - |
| Performance IQ | 9.6E-01 | -0.003 (-0.105 0.099 ) | rs11690172 | chr2 | 57159959 | A | 41.8 | AC009406.1,AC009406.2 | -0.02 (-0.02 -0.01 ) | 2.0E-08 | + |
| Total IQ | 9.6E-01 | -0.002 (-0.075 0.071 ) | rs10496091 | chr2 | 61255126 | A | 32.8 | USP34,RP11-479F13.1,RP11-493E12.2,AHSA2,AC016747.1,C2orf74 | -0.02 (-0.02 -0.01 ) | 5.6E-10 | + |
| Verbal IQ | 9.7E-01 | 0.002 (-0.075 0.079 ) | rs10496091 | chr2 | 61255126 | A | 32.8 | USP34,RP11-479F13.1,RP11-493E12.2,AHSA2,AC016747.1,C2orf74 | -0.02 (-0.02 -0.01 ) | 5.6E-10 | - |
| Performance IQ | 9.7E-01 | 0.002 (-0.076 0.08 ) | rs2431108 | chr5 | 104612267 | T | 28.4 | RP11-6N13.1 | -0.02 (-0.02 -0.01 ) | 5.3E-09 | - |
| Verbal IQ | 9.7E-01 | 0.002 (-0.077 0.081 ) | rs6799130 | chr3 | 161130013 | C | 48.7 | NMD3,B3GALNT1,PPM1L,RP11-479I16.2 | -0.02 (-0.02 -0.01 ) | 2.8E-08 | - |
| Total IQ | 9.8E-01 | -0.002 (-0.093 0.089 ) | rs572016 | chr12 | 120841280 | A | 45.5 | SPPL3,ARF1P2,CLIC1P1,RPL12P33,RP11-173P15.7 | -0.02 (-0.02 -0.01 ) | 3.5E-08 | + |
| Performance IQ | 9.9E-01 | -0.002 (-0.114 0.11 ) | rs12969294 | chr18 | 37606159 | A | 36.3 | CELF4,MIR4318,RP11-19F9.1 | -0.02 (-0.02 -0.01 ) | 7.2E-09 | + |
| Total IQ | 9.9E-01 | 0.001 (-0.058 0.06 ) | rs2431108 | chr5 | 104612267 | T | 28.4 | RP11-6N13.1 | -0.02 (-0.02 -0.01 ) | 5.3E-09 | - |
| Total IQ | 1.0E+00 | 0.001 (-0.065 0.067 ) | rs324886 | chr5 | 88600784 | T | 41.8 | LINC00461,CTC-470C15.1,MIR9-2,CTC-467M3.1,CTC-467M3.3,CTC-467M3.2 | -0.01 (-0.02 -0.01 ) | 1.9E-08 | - |
